# Downregulation by *CNNM2* of *ATP5MD* expression in the 10q24.32 schizophrenia-associated locus involved in impaired ATP production and neurodevelopment

**DOI:** 10.1038/s41537-021-00159-y

**Published:** 2021-05-21

**Authors:** Zhongju Wang, Yongchang Zhu, Linyan Ye, Qiyang Li, Bo Guo, Hao Zhao, Xiuqin Bao, Qiqi Zhuo, Tengfei Yang, Zhaoqiang Li, Shufen Li, Bingtao Hao, Cunyou Zhao

**Affiliations:** 1 Department of Medical Genetics, School of Basic Medical Sciences, Guangdong Technology and Engineering Research Center for Molecular Diagnostics of Human Genetic Diseases, and Guangdong Engineering and Technology Research Center for Genetic Testing, Southern Medical University, Guangzhou, China; 2 Key Laboratory of Mental Health of the Ministry of Education, Guangdong-Hong Kong-Macao Greater Bay Area Center for Brain Science and Brain-Inspired Intelligence, and Guangdong Province Key Laboratory of Psychiatric Disorders, Southern Medical University, Guangzhou, Guangdong China; 3 Institute of Cancer Research, School of Basic Medical Sciences, Southern Medical University, Guangzhou, Guangdong China

**Keywords:** Schizophrenia, Genetics of the nervous system

## Abstract

Genome-wide association studies (GWAS) have accelerated the discovery of numerous genetic variants associated with schizophrenia. However, most risk variants show a small effect size (odds ratio (OR) <1.2), suggesting that more functional risk variants remain to be identified. Here, we employed region-based multi-marker analysis of genomic annotation (MAGMA) to identify additional risk loci containing variants with large OR value from Psychiatry Genomics Consortium (PGC2) schizophrenia GWAS data and then employed summary-data-based mendelian randomization (SMR) to prioritize schizophrenia susceptibility genes. The top-ranked susceptibility gene *ATP5MD*, encoding an ATP synthase membrane subunit, is observed to be downregulated in schizophrenia by the risk allele of *CNNM2*-rs1926032 in the schizophrenia-associated 10q24.32 locus. The *Atp5md* knockout (KO) in mice was associated with abnormal startle reflex and gait, and *ATP5MD* knockdown (KD) in human induced pluripotent stem cell-derived neurons disrupted the neural development and mitochondrial respiration and ATP production. Moreover, *CNNM2*-rs1926032 KO could induce downregulation of *ATP5MD* expression and disruptions of mitochondrial respiration and ATP production. This study constitutes an important mechanistic component that links schizophrenia-associated *CNNM2* regions to disruption in energy adenosine system modulation and neuronal function by long-distance chromatin domain downregulation of *ATP5MD*. This pathogenic mechanism provides therapeutic implications for schizophrenia.

## Introduction

**Fig. 1:**
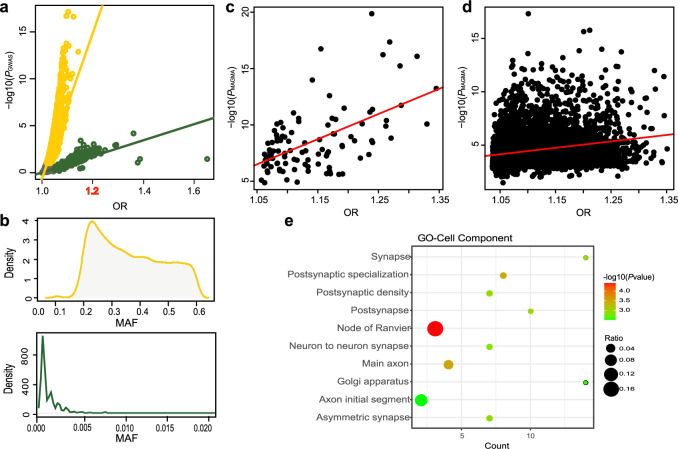
Region-based MAGMA analysis in identifying loci containing OR >1.2 SNPs. **a** Positive linear regression in each two-cluster pair (yellow for variants with MAF >0.1 and green for variants with MAF <0.005) was observed between the −log_10_(*P*_GWAS_) and odds ratio (OR) of the tag-SNPs in each of 108 schizophrenia-associated loci from the PGC2 clump summary data. **b** MAF of SNPs from each of the two clusters in panel **a** are shown by density. **c**, **d** Positive linear regression was observed between region-based MAGMA *P* values (−log_10_(*P*_MAGMA_) and largest ORs in each of 108 PGC2 reported schizophrenia risk loci (*P* = 1.1e-9, *r* = 0.56; **c**), and in each of 5216 200-kb loci (*P* < 2.2e-16, *r* = 0.23; **d**). **e** Gene Ontology (GO) cell component enrichment analysis of 45 MAGMA risk loci that have not reported in the previous study.

Schizophrenia is a chronic, severe, and disabling mental illness that affects ~1% of the population worldwide. It is a highly heritable psychiatric disorder with a complex genetic architecture including contributions from thousands of common and rare variations. Over the past decade, genome-wide association studies (GWAS) research has accelerated the discovery of many genetic loci associated with schizophrenia and has substantially advanced our understanding of these disease^1^. The evidence to date suggests that many risk alleles for common schizophrenia-associated genetic variants may be shared across ancestry groups, but others may be population-specific because of differing causal variants, minor allele frequency (MAF), or linkage disequilibrium (LD) patterns specific to populations of different ancestries^2^. Recently, the largest GWAS from the Psychiatry Genomics Consortium (PGC2) Schizophrenia Working Group identified 108 genome-wide significant loci^1^. However, most risk variants in schizophrenia-associated genetic loci have small individual odds ratio (OR) value, and only two independent loci had index single nucleotide polymorphisms (SNPs) with OR >1.2^3–5^ (Fig. 1a, b), suggesting that more functional risk variants are unidentified and that the genes or functional DNA elements through which the genetic variants identified from GWAS exert their effects on diseases remain largely unknown. Polygenic risk analysis, including gene and gene-set analyses have been suggested as potentially more powerful alternatives to the typical single-SNP analyses performed in a GWAS, especially for studying polygenic traits^6^. Recent approaches, such as multi-marker analysis of genomic annotation (MAGMA)^7^, address these issues by using a multiple regression model to properly incorporate LD between markers and to detect multi-marker effects and identify additional genes and gene sets associated with complex genetic diseases. As significantly positive correlation was observed between region-based MAGMA *P* value and the largest OR in each of PGC2 identified 108 schizophrenia-associated loci (Pearson *P* = 1.09e-9 and *r* = 0.56, Fig. 1c), as well as in PGC GWAS data for major depression and bipolar disorders (Supplementary Fig. 1)^8,9^, we raised the hypothesis that region-based multi-SNP integrative MAGMA analysis can help identify schizophrenia-associated loci containing variants with OR >1.2 based on the PGC2 schizophrenia GWAS data^1^. We also identified a potential specific molecular mechanism of genetic risk related to the 10q24.32 locus and its long-distance regulation of *ATP5MD* expression, which is involved in impairments of energy metabolism and neurodevelopment underlying the risk of schizophrenia.

## Results

### Applying a MAGMA approach to identify schizophrenia risk loci containing OR >1.2 SNPs

To identify more risk loci containing SNPs with OR >1.2, we therefore conducted genome-wide region-based MAGMA analysis of the PGC2 schizophrenia GWAS data^1^. First, we screened all subthreshold surpassing SNPs (*P* < 0.001) and reordered these SNPs with respect to their OR value from the largest to the smallest ORs, and identified 5216 200-kb loci, each of which contains an SNP showing the largest OR value (index SNP) within the 200-kb locus ranging from 100 kb upstream to 100 kb downstream of the index SNP. Among these index SNPs, (521) 10% of the index SNPs showed OR >1.2. Next, we conducted genome-wide region-based MAGMA association analysis with SNPs (*P* < 0.001) within each of 5216 200-kb loci and identified that 336 risk loci passed the genome-wide significance test (*P*_MAGMA-PGC2_ < 5e-08; Dataset 1 and Supplementary Fig. 2). Furthermore, we performed MAGMA analysis of genome-wide 5216 loci with PGC3 schizophrenia GWAS data^10^ and observed that 276 of 336 PGC2 MAGMA risk loci passed the genome-wide significance test in PGC3 MAMGA analysis, and all the top 10 PGC2 MAGMA risk loci remained significant in PGC3 dataset (Dataset 1). Intriguingly, a positive correlation was also observed between region-based MAGMA *P* value and the largest OR value in each of 5216 loci (Pearson *P* < 2.2e-16 and *r* = 0.23, Fig. 1d), indicating that region-based multi-SNP integrative MAGMA analysis can help identify risk loci containing variants with OR >1.2.

We then evaluated the credibility of our 336 risk loci identified by MAGMA analysis with the reported schizophrenia GWAS datasets, specifically, the PGC2, CLOZUK, Chinese *Han*, and PGC3 datasets^1,11,12^. Of the 336 loci, 223 loci are located within 500 kb around the genome-wide significant regions previously reported in the PGC2 GWAS dataset (Dataset 1 and Supplementary Fig. 3a)^1^; examples include the reported *AS3MT* and *NT5C2*, which were top ranking in the 10q24.32 locus^13,14^. Of 113 loci that were not reported in the PGC2 GWAS dataset, 68 loci passed genome-wide significance (*P* < 5e-08) in meta-analysis of CLOZUK, Chinese *Han*, or PGC3 GWAS datasets (Supplementary Fig. 3b, c)^11,12^. Moreover, 45 out of the 336 MAGMA loci have not been previously implicated in schizophrenia PGC2, CLOZUK, Chinese *Han*, or PGC3 GWAS datasets, and showed significantly enrichment in neuron or synapse associated pathways through Gene Ontology (GO) using ToppFun^15^ (Fig. 1e). We also found genes implicated in 45 loci significantly enriched in previously reported schizophrenia-associated gene sets, such as fragile X mental retardation protein (FMRP) and postsynaptic density (PSD) gene sets^16–21^ (Supplementary Table 1). The above evaluations demonstrate the effectiveness of risk loci identified by the multi-SNP integrative MAGMA approach in identifying potential schizophrenia risk genes.

### SMR integrative analysis identified *ATP5MD* as a susceptibility gene linked with the 10q24.32 risk locus

To gain further insights into the biological roles of MAGMA identified schizophrenia risk loci, we then conducted multi-SNP based summary-data-based mendelian randomization (SMR) SMR^22,23^ analysis integrating schizophrenia PGC2 GWAS^1^ and LFuN brain expressional quantitative trait loci (eQTL)^24^ to identify the target genes whose mRNA levels were affected by our MAGMA-identified risk loci. We prioritized that four susceptibility genes, including *GLT8D1*, *ATP5MD5*, *SCFD1*, and *KCNJ13*, whose expression levels were significantly associated with genetic variants of the MAGMA-identified risk loci passed Bonferroni adjustment (*adj.P*_SMR_multi_ < 0.05, Table 1 and Dataset 2). We further performed meta-analysis of differentially expressed genes (DEG) in the prefrontal cortex (PFC) brain tissues of 286 schizophrenia cases versus 343 nonpsychiatric controls from the Gene Expression Omnibus (GEO) dataset^25–33^, and observed that the mRNA expression of *ATP5MD5* was significantly lower in PFC brain tissues of schizophrenia cases compared with nonpsychiatric controls (*P*_meta-DEG_ = 3.96e-3; Supplementary Fig. 4). However, the mRNA levels of *GLT8D1*, *SCFD1*, and *KCNJ13* did not differ between schizophrenia and controls in this meta-analysis (*P*_meta-DEG_ > 0.2; Dataset 2). Furthermore, altered *ATP5MD* expression in schizophrenia was also observed in the Common Mind Consortium (CMC, minimal *P* = 0.005)^34^ and in the Lieber Institute for Brain Development (LIBD, minimal *P* = 0.008)^35^ datasets (Dataset 3).

**Table 1:** Significant target genes identified in multi-SNP-based integrative analysis.

GWAS loci location (hg19)	Gene symbol	Index SNP	OR	*P*_GWAS_	Region *P*_MAGMA-PGC2_	Target gene	Distance to index SNP (Mb)	*P*_SMR_	*P*_SMR-multi-_	*adj.P*_SMR-multi-_	*P*_meta-DEG_
Chr.3: 52793343- 52993343	*NEK4; ITIH1; ITIH3; ITIH4; RP5-966M1.6; MUSTN1; TMEM110-MUSTN1; TMEM110; SFMBT1*	rs6765170	1.19	2.94e-06	2.04e-10	*GLT8D1*	0.153	8.16e-09	1.44e-08	1.42e-05	4.02e-01
Chr.10: 104756100-104956100	*CNNM2; NT5C2*	rs77593808	1.21	1.55e-05	1.79e-16	*ATP5MD*	0.300	3.39e-03	5.78e-08	5.69e-05	3.96e-03
Chr.10: 104638032-104838032	*BORCS7-ASMT; AS3MT; CNNM2*	chr10_104738032_D	1.23	1.14e-05	2.40e-14	*ATP5MD*	0.418	5.59e-03	6.93e-07	6.82e-04	3.96e-03
Chr.10: 104922649-105122649	*NT5C2; RPEL1; INA; PCGF6*	rs188911457	1.27	7.29e-05	1.07e-14	*ATP5MD*	0.134	3.11e-04	1.20e-06	1.18e-03	3.96e-03
Chr.10: 104484108- 104684108	*SFXN2; WBP1L; CYP17A1; BORCS7; BORCS7-ASMT; AS3MT; CNNM2*	rs147010054	1.20	8.06e-04	1.48e-16	*ATP5MD*	0.572	3.29e-03	1.34e-06	1.32e-03	3.96e-03
Chr.3: 52165321- 52365321	*POC1A; ALAS1; TLR9; TLR9; TWF2; PPM1M; WDR82; GLYCTK; DNAH1*	rs115585422	0.88	9.33e-05	1.88e-09	*GLT8D1*	0.475	5.55e-06	1.39e-06	1.37e-03	4.02e-01
Chr.3: 52950748- 53150748	*SFMBT1; RP11-894J14.5; RFT1*	rs77432708	1.15	1.55e-04	2.73e-09	*GLT8D1*	0.311	9.19e-06	1.74e-06	1.71e-03	4.02e-01
Chr.3: 53075017-53275017	*SFMBT1; RP11-894J14.5; RFT1; PRKCD; TKT*	rs1080500	1.07	1.85e-09	6.59e-10	*GLT8D1*	0.435	3.08e-05	6.23e-06	6.13e-03	4.02e-01
Chr.14: 30170236-30370236	*PRKD1*	rs111997347	0.82	8.63e-04	1.20e-10	*SCFD1*	0.821	2.49e-04	9.61e-06	9.46e-03	1.94e-01
Chr.2: 233546728-233746728	*EFHD1; GIGYF2; KCNJ13; C2orf82; NGEF*	rs283483	0.89	1.37e-04	2.41e-11	*KCNJ13*	0.005	1.24e-02	3.08e-05	3.03e-02	6.07e-01

Target genes of the MAGMA risk loci based on the *adj.P*_SMR-multi_ < 0.05 were identified by multi-SNP based SMR analysis.

*P*_SMR_ is shown for the *p* value from the single SNP based SMR analysis. *P*_SMR-multi_ is shown for the *p* value from the multi-SNP based SMR analysis. *adj.P*_SMR-multi_ is shown as Bonferroni adjustment for *P*_SMR-multi_.

The location of GWAS risk loci is shown with its coordinate in the corresponding chromosome built on hg19.

The protein-coding gene symbol is shown for genes annotated for the corresponding loci identified by multi-SNP MAGMA analysis.

Index SNP is shown for the SNP with the largest odds ratio (OR) in the MAGMA-identified schizophrenia risk GWAS loci, and the GWAS *p* value of each index SNP from PGC2 is indicated by *P*_GWAS_.

The distance to the index SNP is shown for the distance (Mb) from the target gene to the index SNP.

*P*_meta-DEG_ from meta-analysis of schizophrenia-associated brain DEG from nine datasets.

**Fig. 2:**
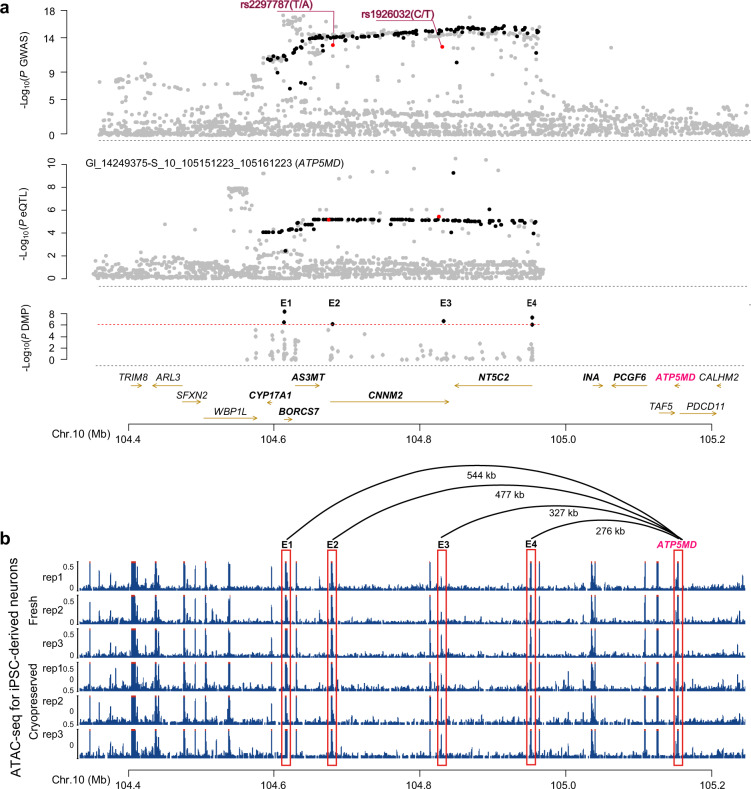
Effects of 10q24.32 locus on the *ATP5MD* expression. **a** Matched association patterns among GWAS association (top), *ATP5MD* eQTL (middle) and methylation of DMP (bottom) in E1, E2, E3, and E4 in chr10: q24.32. SNPs with GWAS *P* < 5e-8 and ATP5MD-eQTL *P* < 1e-3 were identified as colocalized SNPs shown with back or red dots, and non-colocalized SNPs shown with gray dots. **b** ATAC-seq data for human iPSC-derived neurons are shown for the same chromosome regions as in panel **a**. Plot were generated with WashU Epigenome Brower (http://cistrome.org/browser). Enhancer region E1 (chr10:104611780-104613953, hg19), E2 (chr10:104678717-104680610), E3 (chr10:104829163-104830971), E4 (chr10:104880067-104881657), and *ATP5MD* (chr10:105,148,809-105,156,270) were highlighted in red rectangles. Distance between *ATP5MD* and E1–E4 were also labeled.

*ATP5MD*, also named *USMG5* (upregulated during skeletal muscle growth protein 5), encodes the ATP synthase membrane subunit and also known as diabetes mellitus–associated protein in insulin sensitive tissues, or *DAPIT*^36^. *ATP5MD* displayed the remarkable multi-SNP-based SMR *P* value, which is 200–5000 times stronger than that of the single-SNP-based SMR *P* value (Table 1). This finding is also validated by matched colocalized signatures (Fig. 2a, Supplementary Table 2, and Supplementary Fig. 5) between the *ATP5MD* eQTL and GWAS associations of more than two genes; such as association including the uncharacterized *CNNM2*, and the previously reported *AS3MT* and *NT5C2*, which are in the 10q24.32 locus^1,13,14,37^. Co-expression analysis further demonstrated that the *ATP5MD* expression level was correlated with the expression levels of *CNNM2* (*r* = −0.62, *P* = 3e-49), *NT5C2* (*r* = −0.38, *P* = 4.91e-19), *WBP1L* (*r* = −0.43, *P* = 4.38e-22), and *SFXN2* (*r* = −0.38, *P* = 5.98e-17) located in 10q24.32 locus based on LIBD RNA-seq dataset (Supplementary Table 3 and Supplementary Fig. 6). These schizophrenia association signatures were also validated in three other independent schizophrenia GWAS datasets (Chinese *Han*, PGC bipolar disorder and schizophrenia, and CLOZUK GWAS)^11,12,38^, as well as in another independent LIBD brain eQTL dataset (Supplementary Fig. 7)^35^. The effect of this locus on the risk of schizophrenia may be mediated by the expression regulation of multiple genes, including the uncharacterized *ATP5MD* and the previously reported *AS3MT* and *NT5C2*^13,14,37^, thus conferring a risk for schizophrenia.

### Long-distance downregulation of *ATP5MD* expression by *CNNM2* involved in impairments of ATP production and neurodevelopment

**Fig. 3:**
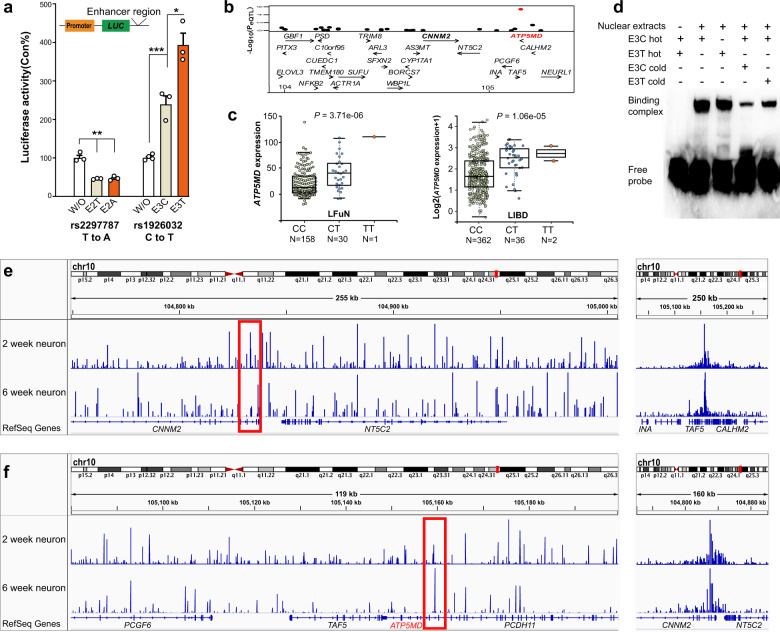
Regulatory role of *CNNM2*-E3 in the *ATP5MD* expressoin. **a** Effects of the potential enhancer/silencer E2 and E3 regions on *ATP5MD* promoter activities of luciferase reporter in SK-N-SH cells. W/O, the reporter without an enhancer/silencer; E3 contains a C allele for E3C or a T allele for E3T in rs1926032(C/T); E2 contains a T allele for E2T or an A allele for E2A in rs2297787 (T/A). A two-sided *t* test was used for comparisons between the two indicated groups (*P < 0.05, **P < 0.01, and ***P < 0.001). The data were shown as the mean ± SEM from at least three independent experiments with duplications. **b** Association of the rs1926032(C/T) genotype with 26 gene expression in the 10q24.32 locus in the PFC from the LFuN dataset (*n* = 189). **c** Association of the rs1926032 (C/T) genotypes with *ATP5MD* expression in the PFC sample from the LFuN dataset (left) or the LIBD datasets (right). *P* values were calculated by MatiexEQTL using expression for the LFuN dataset or log2 (expression +1) for the LIBD dataset. **d** EMSA competition analysis was performed with hot E3C or E3T probes and cold E3C or E3T probes as competitors with HEK 293 T cell nuclear extracts. DNA binding complex bands and a free probe band are indicated in the image. **e**, **f** The interactions of 4 C data from the *ATP5MD* promoter (**e**) and the *CNNM2*-E3 (**f**) viewpoint in 2-week and 6-week iPSC-derived neurons. The red frames indicate the *CNNM2*-E3 region in panel (**e**) and *ATP5MD* promoter region in panel (**f**).

Since the four index SNPs showing the largest OR value in the given MAGMA loci have low genotype frequency and didn’t surpassed the genome-wide significant level (*P* > 5e-8) in PGC2 schizophrenia GWAS dataset and didn’t display significant eQTL (*P* > 1e-3) in LIBD dataset or LFuN dataset (Supplementary Fig. 8), we then used DNA methylation data from PFC brain tissues of 191 schizophrenia patients and 335 nonpsychiatric controls in the LIBD dataset (GSE74193)^39^ to identify the functional regions of the identified 10q24.32 locus with a role in regulating of *ATP5MD* expression. We screened four schizophrenia-associated differential methylation probe (DMPs) (*P* < 1e-6; the four DMPs located region named E1, E2, E3, and E4 in Fig. 2a bottom) within the identified risk 10q24 locus. Furthermore, we also observed that E1, E2, E3, and E4 regions were enriched in activate chromosome states from roadmap 15 DLPFC derived chromosome states (Supplementary Fig. 9)^40^. We next verified their regulatory roles by cloning these four regions into the enhancer region of a luciferase reporter, and observed that the E2 region inhibited *ATP5MD* promoter activities, whereas the E3 region remarkably enhanced *ATP5MD* promoter activity in human SK-N-SH neuroblastoma cell line (Fig. 3a and Supplementary Fig. 10). These results, together with the active chromatin states observed in the E2 and E3 regions from human neuron assay for transposase-accessible chromatin using sequencing (ATAC-seq) datasets (Fig. 2b)^41^, suggest that the E2 and E3 regions might contain functional elements necessary for the regulation of *ATP5MD* expression.

We further screened a schizophrenia risk eQTL SNPs in E3 region (rs1926032C/T, GWAS *P* = 2.69e-13; Fig. 2a) that only correlated with *ATP5MD* expression (Fig. 3b), showing downregulated *ATP5MD* expression by the risk C allele of rs1926032 in both the LFuN (eQTL *P* = 3.71e-6; *n* = 189) and LIBD (eQTL *P* = 3.71e-6; *n* = 400) brain eQTL datasets (Fig. 3c)^24,35^. This C allele-dependent *ATP5MD* downregulation were further validated in luciferase reporter assay (Fig. 3a, left), in which the *ATP5MD* promoter activity for the enhancer containing the risk C allele was significantly lower than that of the T allele in SK-N-SH cell line. Although there is a schizophrenia risk eQTL SNPs in E2 (rs2297787, GWAS *P* = 1.5e-13, eQTL *P* = 7e-06; Supplementary Fig. 11) region that correlated with *ATP5MD* expression, we didn’t observe genotypic effect on the *ATP5MD* promoter activity in luciferase reporter assay (Fig. 3a, right). Moreover, the risk C allele of rs1926032 in E3 also displayed a higher binding activity than the T allele did as demonstrated by electrophoretic mobility shift assay (EMSA) with the human embryonic kidney 293 T (HEK293T) nuclear extracts (Fig. 3d). Finally, we also observed a significant interaction between the E3 region and *ATP5MD* as revealed by the circularized chromatin conformation capture (4 C) assay in human induced pluripotent stem cells (iPSC)-derived cortical neurons (Fig. 3e, f), and E3 region was also annotated to *CNNM2* based on activity-by-contact method with superior temporal gyrus and entorhinal cortex Hi-C data^42,43^, supporting a key regulatory role of the rs1926032-containing E3 region located in *CNNM2* in the *ATP5MD* expression.

**Fig. 4:**
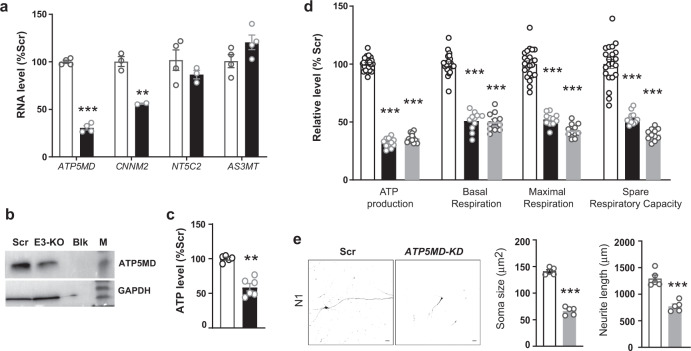
Effect of *ATP5MD* downregulation on mitochondrial respiration and neurodevelopment. **a**–**c** Effects of KO rs1926032-containing E3 region (black column) on the RNA expression levels of *ATP5MD*, *CNNM2*, *NT5C2*, and *AS3MT* (**a**), ATP5MD protein level (**b**), and intracellular ATP level (**c**) in SK-N-SH cells. **d** Effects of KO rs1926032-containing E3 region (black) or *ATP5MD*-KD (gray) in relative to the controls (white) on the ATP production, basic respiration, maximal respiration, and spare respiratory capacity of mitochondria in iPSC-derived neurons. **e** *ATP5MD-*KD attenuated neural development of iPSC-derived neurons. Sample projected confocal images (left) of 2-week-old human iPSC-derived cortical neurons with lentivirus-mediated coexpression of GFP and shRNA-control (Scr, white column) or shRNA-*ATP5MD* (gray column). Quantifications of soma size and neurite length were obtained from one of three independent iPSC-derived neuron samples (N1). The scale bar represents 20 μm. The data were shown as the mean ± SEM from at least two independent experiments with duplications. A two-sided *t* test was used for comparisons between the two indicated groups (*P < 0.05, **P < 0.01, and ***P < 0.001).

To further validate the regulatory role of rs1926032-containing E3 region in *ATP5MD* expression, we next used the CRISPR-Cas9 system to KO the rs1926032-containing E3 region (Supplementary Fig. 12) in the SK-N-SH cell line and human iPSC-derived cortical neurons to verify whether KO would affect *ATP5MD* expression and its function. We observed that the *ATP5MD* and *CNNM2* mRNA levels decreased significantly in the rs1926032-KO SK-N-SH cells compared to the controls (Fig. 4a). We then observed that the rs1926032 disruption could reduce the ATP5MD protein level (Fig. 4b) and intracellular ATP level (Fig. 4c) compared to those of the controls. We also observed that rs1926032 disruption in human iPSC-derived cortical neurons could reduce ATP production and mitochondrial respiration compared to those of the controls (Fig. 4d). *ATP5MD* is present in oligodendrocytes, oligodendrocyte precursor cells (OPCs), neurons, microglia, and astrocytes^44^, and it is highly expressed in human PFC brain tissues (Supplementary Fig. 13)^45^, showing a gradually increasing expression in the earlier developmental stage from the BrainSpan dataset^46^. Tissue-specific expression pattern of *CNNM2* was also observed from GTEx dataset (Supplementary Fig. 14)^45^. Since *ATP5MD* mRNA expression was observed to be downregulated in schizophrenia (Supplementary Fig. 4), we therefore employed shRNA to directly knockdown (KD) the endogenous *ATP5MD* expression, and observed that *ATP5MD-*KD significantly attenuated neural development, yielding a decreased soma size and neurite length of 2-week-old human iPSC-derived cortical neurons (one-way ANOVA *P* < 1e-4. Figure 4e and Supplementary Fig. 15); *ATP5MD-*KD in human iPSC-derived cortical neurons also reduced mitochondrial respiration and ATP production (Fig. 4d), as well as intracellular ATP level (Supplementary Fig. 16) compared to that of the controls. More importantly, *atp5md*-KO mice were reported to display abnormal brain-related behaviors including abnormal startle reflex and gait (Supplementary Fig. 17), as shown by the International Mouse Phenotyping Consortium (IMPC) dataset^47^. Altogether, these results suggested that impaired ATP production and neurodevelopment induced by *CNNM2*-rs1926032 genotype-dependent *ATP5MD* downregulation might increase susceptibility to schizophrenia.

## Discussion

The present study employed a multi-SNP integrative strategy to help identify risk loci containing OR >1.2 SNPs, and it followed with the prioritization of schizophrenia susceptibility genes in 10q24.32 GWAS locus associated with impaired energy production and neurodevelopment through long-distance downregulation of *ATP5MD* expression. This molecular pathological mechanism that links schizophrenia-associated genetic variants to disruption in energy metabolism and neurodevelopment provides therapeutic implications for schizophrenia.

Our study applied a multi-SNP integrative MAGMA strategy to minimize the interference of MAF and LD degree of genetic variants. We identified 336 schizophrenia risk loci, 68 of which contain OR >1.2 genetic variants and 45 of which are previous not-reported loci involving in neuron and synaptic associated pathways. We also found these genes were significantly enriched in FMRP and PSD gene sets, which have been previously reported implicated in schizophrenia^1,48,49^, demonstrating the effectiveness of our multi-SNP integrative MAGMA strategy in identify schizophrenia risk loci and genes. Of the four schizophrenia-associated target genes identified by SMR, *GLT8D1* has been reported to be associated with schizophrenia and implicated in synapse function^50^, *KCNJ13* has been reported to be associated with neuronal excitability^51^, and *ATP5MD* expression downregulated in PFC brain tissues of schizophrenia patients in our meta-analysis was also observed in PsychEncode consortium (*P* = 0.019) at transcript level^52^. *Atp5md* knock-out mice also showed abnormal startle reflex in acoustic startle and pre-pulse inhibition (PPI) testing^47^, supporting *ATP5MD* as a schizophrenia susceptibility gene. *ATP5MD* gene expression was further predicted to be long-distance regulated by 10q24.32, which is one of the top-ranked MAGMA risk loci identified in our study, containing several schizophrenia risk variations located in *CNNM2*, *NT5C2*, and *AS3MT*. Usually, the genes in closet physical proximity to top risk variants are considered to be the most likely susceptibility genes. Our finding that schizophrenia risk *ATP5MD* is located in 200–600 kb downstream of the index SNP of 10q24.32 GWAS loci, together with previous reports that *NT5C2*, *AS3MT*, and *BORCS7* in this locus have been reported as schizophrenia risk genes^1,13,37^, support that susceptibility gene expression could be regulated by risk genetic variants through either close range or long-distance chromatin interactions.

We provided multiple lines of data including DNA methylation, chromatin conformation (4 C), eQTL, GWAS association data, luciferase reporter, and EMSA to support the role of *CNNM2* in regulation of *ATP5MD* expression through long-distance regulation. In particular, the risk allele of *CNNM2*-rs1926032 (C/T) association with schizophrenia from PGC, Chinese *Han*, and CLOZUK GWAS data^10–12,38^, was correlated with the downregulation of *ATP5MD* expression in human DLPFC brain tissues of the LFuN and LIBD eQTL datasets^24,35^ and luciferase reporter gene. These results were consistent with the observation of the declined *ATP5MD* mRNA level in the PFC brain tissues of schizophrenia patients^25–33^. Previously, ATP5MD was reported to be associated with white matter hyperintensities (WMH)^53^, which have been associated with both schizophrenia and mood disorders, particularly bipolar disorder^54^; a recurrent *ATP5MD* splice-site founder mutation in the Ashkenazi Jewish population have been report to impair mitochondrial complex V dimerization and ATP synthesis^55^. In fact, ATP5MD, a component of an ATP synthase complex^36,55^, is involved in ATP synthesis in mitochondria and displays a gradually increasing expression in the earlier developmental stage from the BrainSpan dataset^46^. Our study demonstrated that *ATP5MD-KD* induced impaired neurodevelopment, and mitochondrial respiration and ATP production. Intriguingly, knocking out the rs1926032-containing *CNNM2*-E3 region also reduced ATP5MD expression and mitochondrial respiration and ATP production, providing further evidence to support the regulatory roles of *CNNM2* in *ATP5MD* expression and energy metabolism.

Much data indicated that psychiatric disorders may be related to a deficit in adenosine system^56–59^, such as recently reported missense variants in *ATP1A3* associated with behavioral disorders and childhood-onset schizophrenia^60^. Intriguingly, the CNNM2 protein is an evolutionarily conserved Mg^2+^ transporter that contains ATP binding domains. ATP can bind to CNNM2 in a manner dependent on the presence of Mg^2+^, which mostly forms complex with Mg^2+^ in cells^61^. Previously, loss of CNNM2 was associated with human intellectual disability^62^, and a truncated allele was found in schizophrenia patients through large-scale exome analysis^63^. In addition, the NT5C2 enzyme encoded by the psychiatric risk gene *NT5C2* in the 10q24.32 locus also has a high affinity for AMP and regulated AMP-activated protein kinase (AMPK) signaling^37^. Together with reports that *CNNM2*, *NT5C2, AS3MT*, and *BORCS7* in the 10q24.32 locus are also involved in the brain development^13,37,62,64^, these results constitute important mechanistic components that link schizophrenia-associated genetic variants in the 10q24.32 locus to the impairments in adenosine system modulates and then to the profound effects on neuronal function such as highly energy consuming neurodevelopment process.

Although our studies identified *CNNM2-*rs1926032 as a *cis*-element that is involved in the regulation of *ATP5MD* through long-distance interactions, we also observed that CTCF and myocyte enhancer factor 2A (MEF2A) showed significant enrichments in the rs1926032-containing E3 region in *CNNM2*, as demonstrated by ChIP-seq from ENCODE datasets^65,66^. CTCF is involved in transcriptional regulation by binding to chromatin insulators and mediating long-range promoter–enhancer/silencer interactions^67^. MEF2A is a DNA-binding transcription factor that activates many muscle-specific and growth factor-induced genes and is involved in muscle development, neuronal differentiation, cell growth control, and apoptosis^68,69^. We observed that rs1926032 displayed genotype-dependent binding differences in EMSA, whether CTCF and MEF2A were enriched at the rs1926032 allele in the E3 region resulting in allelic biased regulation of *ATP5MD* expression remains to be further investigated.

In conclusion, we employed a multi-SNP integrative strategy to help identify additional risk loci containing genetic variants with OR >1.2 and delineated the mechanistic insight gained from the most significant schizophrenia-associated 10q24.32 GWAS loci. Theses loci are involved in disruption of energy metabolism and neurodevelopment; it regulated by the *CNNM2-*rs1926032 risk allele-dependent long-distance downregulation of *ATP5MD* expression in this locus. *Atp5md* knock-out mice also showed abnormal startle reflex, indicating sensory gating deficits of schizophrenia-associated 10q24.32 GWAS loci through long-distance downregulation of energy metabolism associated *ATP5MD* gene. This study has the potential to advance our understanding of common biological pathways contributing to disease, and it provides insights that could accelerate the identification of drug targets and biomarkers for schizophrenia.

## Methods

### MAGMA analysis

Schizophrenia GWAS summary data downloaded from the PGC website^1^ were employed for analysis. We reversed all ORs to 1/OR if OR <1, to make comparison easy. SNPs with the largest OR in each locus were identified as index SNPs. The LD degree (R) was estimated with Pearson’s correlations in LDstore^70^ using genotyping data from the 1000 Genomes Project (1000 GP)^71^ and European ancestry samples as the reference panels. In MAGMA analysis, we first mapped all SNPs (GWAS *P* value < 0.001) in the given loci using a preprocessing annotation step with parameter–annotation, and then we performed MAGMA analysis^7^ with the *P* values of all mapped SNPs and the LD degree in the given locus to obtain a multi-SNP integrative *P* value (*P*_MAGMA_) for that locus. For MAGMA analysis of 108 PGC2 GWAS loci, we mapped all SNPs (GWAS *P* value < 0.001) and the LD degree in each of 108 PGC2 GWAS loci to obtain a *P*_MAGMA_ for that locus. For the genome-wide MAGMA analysis of PGC2 schizophrenia GWAS^1^, we first kept all subthreshold significant SNPs with *P*_GWAS_ < 0.001 and reordered these SNPs with their OR from the largest to the smallest OR value and identified 5216 index SNPs in the given 200-kb locus from 100 kb upstream to 100 kb downstream of the index SNP. We then mapped all SNPs (GWAS *P* value <0.001) and the LD degree in each of 5216 loci to obtain a multi-SNP integrative *P* value (*P*_MAGMA_ < 5e-08) for the given locus as a schizophrenia risk locus. We also performed MAGMA analysis for these 5216 loci with PGC3 GWAS dataset^10^ to cross-validate the MAGMA results.

We annotated genome-wide 5216 loci with previously reported schizophrenia risk loci, including PGC2, Chinese *Han*, CLOZUK, and PGC3 GWAS datasets^1,10–12^. Loci located within 500 kb around previously reported loci in these three datasets were annotated as previously reported loci. We then performed gene annotation (only protein-coding genes were annotated) for genome-wide 5216 loci using BioMart (GRCh7.p13) from the Ensembl website, and performed GO using ToppFun from ToppGene Suite (https://toppgene.cchmc.org/enrichment.jsp)^15^ for genes located within 45 loci out of 336 MAGMA identified loci, with protein-coding genes as background. We also performed enrichment analysis with previously reported schizophrenia-associated gene sets including FMRP targets, PSD proteins, activity-regulated cytoskeleton-associated protein-the *N*-methyl-d-aspartate receptor complex (ARC_NMDAR), genes carrying de novo schizophrenia variants and γ-aminobutyric acid (GABA) gene sets using two-tail Fisher’s exact test^16–21^.

### SMR analysis

To gain further insights into the biological roles of MAGMA identified schizophrenia risk loci, we then conducted multi-SNP based SMR^22,23^ analysis integrating schizophrenia GWAS and brain eQTL to identify the target genes whose mRNA levels were affected by our MAGMA-identified risk loci. We employed brain eQTLs from 193 neuropathologically normal human PFC (LFuN dataset)^24^ for discovery dataset and an independent brain eQTL dataset from the LIBD (from frontal cortex sample of 175 schizophrenia cases and 237 nonpsychiatric controls)^35^ for validation of the interested locus. eQTL analysis was performed with the Package MatrixEQTL^72^ in R with linear regression using sex, age, and pmi et al as covariates. IMPUTE2 was used for the imputation analysis^73^ and European ancestry samples from 1000 GP were used as the reference panel^70^. We employed PGC2 schizophrenia GWAS in SMR analysis for discovery dataset and three independent schizophrenia GWAS datasets: Chinese *Han* GWAS (7699 cases and 18327 controls)^11^, PGC bipolar disorder and schizophrenia GWAS (33,426 cases and 32,541 controls)^38^ and CLOZUK GWAS (11,260 cases and 24,542 controls)^12^, for validation of the interested locus.

In SMR analysis, genes located wtihin 1 Mb around the 200-kb MAGMA risk locus were considered to be potential target genes and included in SMR analysis. We first used the command --make-besd to convert the eQTL data from a Matrix eQTL format into a BESD format, and then we updated the file with the commands --update-epi and --update-esi. Then, we used the command --smr-multi to apply a multi-SNP-based SMR analysis^22^, which combined the information from all variants surpassing the *P* eQTL threshold (*P* < 1.0e-3). Due to the relatively small sample size of the brain eQTL data, we set the *P* eQTL threshold as 1.0e-3 with the parameter --peqtl-smr 1.0e-3. The default parameter of 0.9 for --ld-pruning was used to remove SNPs in very high LD with the top associated variants. Multi-SNP based SMR *P* values were adjusted with Bonferroni method to obtain adj.*P*_SMR-multi_, and genes with adj.*P*_smr-multi_ < 0.05 were identified as target susceptibility genes.

We conducted the meta-analysis with DEG summary data in the PFC from 286 schizophrenia patients and 343 nonpsychiatric controls from nine published papers to examine schizophrenia-associated expression patterns of SMR identified genes^25–33^ using the metafor package in R^74^ showing the effect sizes and 95% confidence intervals (CIs) from each data set and the pooled effects (random-effects model, RE model). The developmental expression profile was obtained from the BrainSpan dataset^46^. The tissue expression profile was obtained from the GTEx project^45^ or from different cell types of the brain.

### Regulatory region screening

We employed public datasets including methylation profiling and ATAC-seq to screen functional regions in regulation of *ATP5MD* expression. Methylation profiling by genome tiling array in PFC, including 225 schizophrenia cases and 450 controls from the LIBD dataset (GSE74193)^39^. We obtained the processed data and performed the DMP analysis with R package limma^75^ using linear regression with sex and race as covariates, and screened DMPs for a candidate enhancer region. We obtained ATAC-seq data from six human iPSC-derived neurons^41^ from Cistrome DB^76,77^, and visualized the data with WashU Epigenome Browser.

### 4 C assay

We also employed 4 C assay with 2-week- and 6-week-old iPSC-derived neurons to further validte the regulatory role of candidate enhancer region in *ATP5MD* expression using previously established protocols^78^. In brief, the chromatin was cross-linked with formaldehyde, followed by two rounds of degestion-ligation using two different 4 bp cutters, MboI, and NlaIII. 4 C templates were then amplified with specific 4C-primers (Supplementary Table 4), which were designed for correponding bait regions. The DNA was sequenced with the 150 bp pair-end sequencing strategy on HiSeq2000 platform. The sequencing data were aligned to the hg19 reference and the generated bedgraph files were used to identify the significant chromatin interaction with bait regions using the R package 4c-ker^79^.

### Dual luciferase reporter assay

To verify the functional region of the identified GWAS loci involved in regulation of *ATP5MD* expression, we cloned E1 (chr10:104611780–104613953, hg19), E2 (chr10:104678717–104680610), E3 (chr10:104829163–104830971), and E4 (chr10:104880067–104881657)-containing schizophrenia-associated DMPs (#cg25193742 and cg10615065 in E1, cg14737131 in E2, cg06694589 in E3, and cg10116432 and cg13215387 in E4) within the identified 10q24 GWAS loci into the enhancer region of a pGL4.11 luciferase reporter (Supplementary Table 4). Mutations at the SNP sites were achieved using the site-directed mutagenesis method by replacing a major allele with a minor allele at SNP sites. These reporter constructs were transiently co-transfected into SK-N-SH cells together with the pRL-TK luciferase plasmid as an internal control for transfection efficiency using the Lipofectamine 2000 reagents. Cells were harvested 48 h after transfection, and the dual luciferase activity (Promega) was measured with the Wallac Victor V 1420 Multilabel Counter (PerkinElmer, San Jose, CA, USA). Three independent experiments were performed.

### EMSA

We employed EMSA with nuclear extracts prepared from the HEK293T cells to evaluate the affinity of rs1926032 C or T allele using a Chemiluminescent Nucleic Acid Detection Module Kit (Thermo Fisher, USA). The reaction samples were run on 6% native polyacrylamide gels in 0.5x Tris/boric acid/EDTA buffer. The binding reactions were transferred to nylon membranes with a Bio-Rad wet transfer apparatus, and DNA was then cross-linked to the membrane with an ultraviolet illuminator. The biotin-labeled DNA (Life Technologies) complexes were visualized and quantified with a chemiluminescence imaging system (Tanon 4200; Tanon, China).

### Mitochondrial stress test and intracellular ATP level measurement

We employed an XF Cell Mito Stress Test kit to assess mitochondrial function by directly measuring the oxygen consumption rate (OCR) of cells in an XF96 extracellular flux analyzer (Seahorse Bioscience, MA, USA). Oligomycin, FCCP, and rotenone were added directly into wells until they reached final concentration of 1 μM Oligo, 0.7 μM FCCP, and 0.5 μM Rotenone, according to the manufacturer’s instructions. Following normalization of the OCR on the protein concentrations measured using a Pierce BCA protein assay kit (Thermo Scientific), ATP production, basal respiration, maximal respiration, and spare respiratory capacity were automatically calculated with supporting software.

We also employed a CellTiter-Glo^®^ Luminescent Cell Viability Assay (G7570, Promega) to measure intracellular ATP levels. Cell lysates were measured with the Wallac Victor V 1420 Multilabel Counter (PerkinElmer, San Jose, CA, USA), and ATP levels were calculated after adjustment by protein concentrations measured using the Pierce BCA protein assay kit.

### Cell, plasmid, and antibody

The human SK-N-SH cell line (ATCC HTB-11) and HEK293T cell line (ATCC CRL-3216) were cultured in Dulbecco’s minimal essential medium (DMEM) supplemented with 10% fetal bovine serum (FBS) and maintained at 37 °C with 5% CO_2_. Cortical neuron differentiation from de-identified human iPSCs purchased from Cellapy Biological Technology (Beijing, China) was adapted from previously established protocol^80^.

*ATP5MD*-KD was performed with lentiviral short hairpin RNA (shRNA) vectors (pLKO.1) purchased from Virgen (China). Small guide (sgRNA) oligonucleotides were designed to target *CNNM2*-E3 region (Supplementary Table 4) and then cloned into a transfer lentiviral vector (lentiCRISPRv2). Transduction of viral particles into SK-N-SH cells or iPSC-derived neurons was performed according to the manufacturer’s protocol. *ATP5MD*-KD was confirmed by qRT-PCR and western blotting with anti-ATP5MD (1:400 Abcam ab108225). *CNNM2*-E3-KO was examined by sequencing the target DNA extracted from the transduced cells.

### Reporting Summary

Further information on research design is available in the Nature Research Reporting Summary linked to this article.

## Supplementary information

Supplementary Information

Supplementary Data 1

Supplementary Data 2

Supplementary Data 3

Reporting Summary

## Data Availability

All data generated in the study are included in the article or uploaded as supplementary materials. Sequencing data of 4 C assay have been deposited to NCBI GEO site with GEO (GSE171077). GWAS summary data were downloaded from the PGC website (http://www.med.unc.edu/pgc), Bio-X institute (http://gwas.bio-x.cn/), and Centre for Neuropsychiatric Genetics and Genomics (https://walters.psycm.cf.ac.uk/). Brain eQTLs data were downloaded from LFuN website (http://labs.med.miami.edu/myers/LFuN/LFUN/INDEX.html) and from LIBD eQTL website (http://eqtl.brainseq.org/phase1/eqtl/). Brain methylation data from LIBD institute were downloaded from GEO (GSE74193). Expression data across different tissues were downloaded from GTEx website (https://www.gtexportal.org/home), and data across development stages were downloaded from BrainSpan (http://www.brainspan.org/static/home). DEG datasets were collected from PsychENCODE (https://www.nimhgenetics.org/resources/psychencode#), LIBD (http://eqtl.brainseq.org/phase1/sz/) and GEO (GSE10784, GSE21935, GSE35977, GSE92538, GSE12649, GSE12654, GSE53987, GSE17612, and GSE21138). ATAC-Seq data were obtained from GEO (GSE78036).

## Code availability

Code used for data processing and analysis is available on request from the corresponding author.
