# Supplementary material for: Downregulation by CNNM2 of ATP5MD expression in the 10q24.32 schizophrenia-associated locus involved in impaired ATP production and neurodevelopment
Source: NPJ Schizophr. 2021 May 21;7:27. doi: 10.1038/s41537-021-00159-y (PMC8139961; doi:10.1038/s41537-021-00159-y)
Supplement: Supplementary file 1 — Supplementary Information [file 41537_2021_159_MOESM1_ESM.pdf]

Supplementary Figures

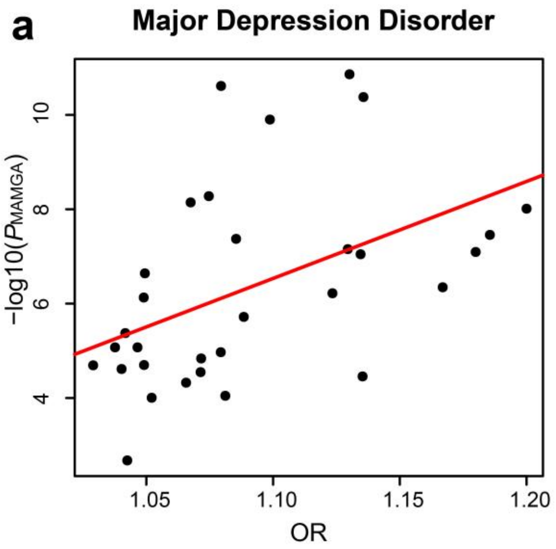

Supplementary Figure 1. Positive linear regression was observed between region-based MAGMA *P* values (-log$_{10}$(P_{MAGMA}) and the largest ORs in each of major depression disorder risk loci (*P*=0.008, r= 0.47; **a**) or in bipolar disorder risk loci (*P*=0.077, r=0.30; **b**).

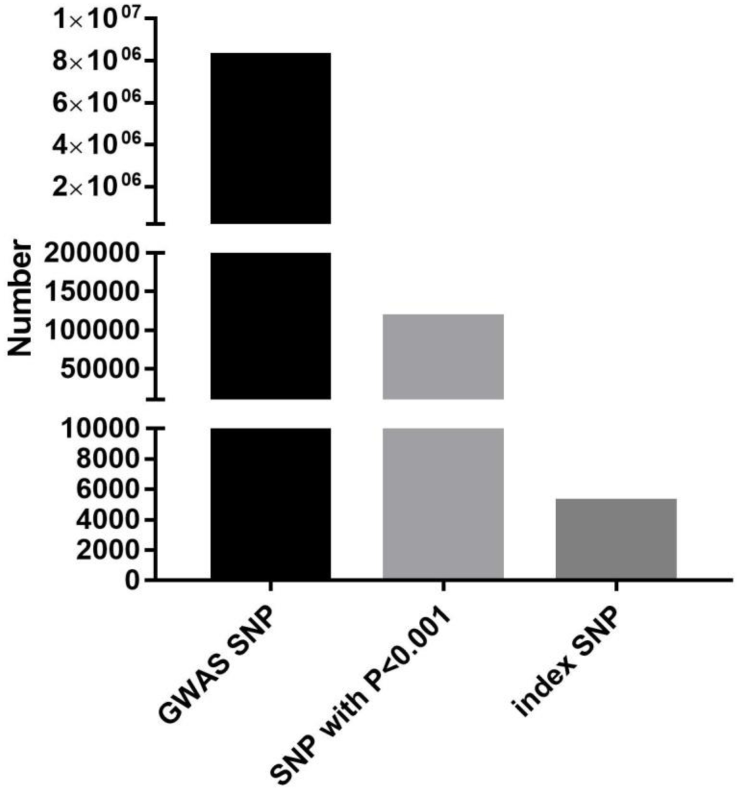

Supplementary Figure 2. Barplot for number of GWAS SNP, SNP with *P*<0.001 and index SNP for MAGMA analysis.

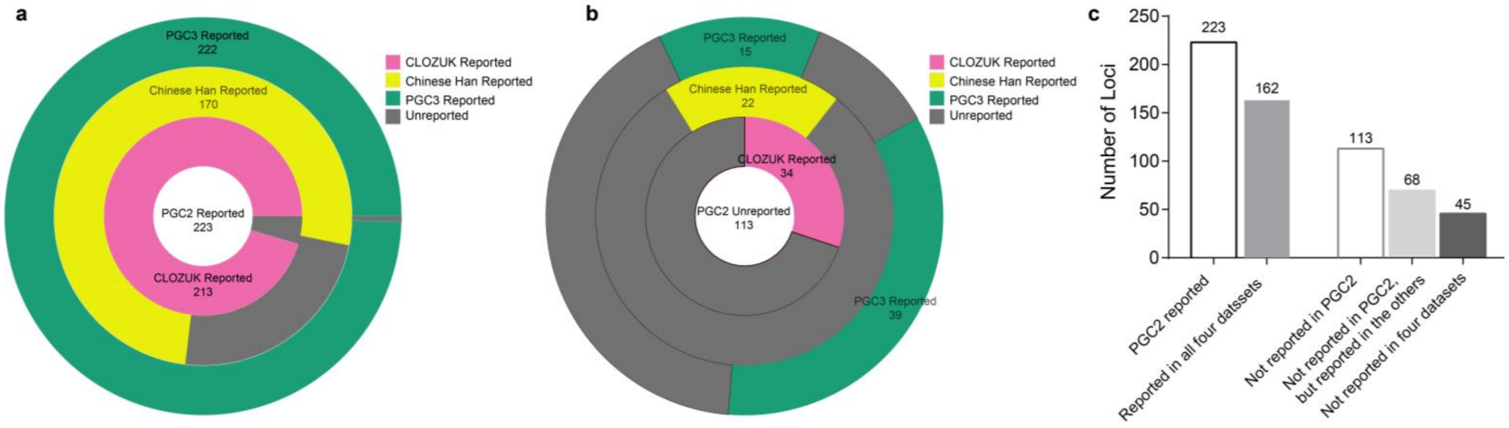

Supplementary Figure 3. (**a**) Pie chart for overlapping between PGC2 reported GWAS loci and three other datasets reported GWAS loci. Of 223 loci, 213 loci were reported in CLOZUK, 170 loci were reported in Chinese *Han* and 222 were reported in PGC3 GWAS datasets. (**b**) Pie chart for overlapping between PGC2 unreported GWAS loci and three other datasets reported GWAS loci. Of 113 MAGMA risk loci, 34 loci were reported in CLOZUK, 22 loci were reported in Chinese *Han* and 54 were reported in PGC3 GWAS datasets. (**c**) Bar plot for MAGMA risk loci overlapping with PGC2, CLOZUK, Chinese Han and PGC3 GWAS risk loci.

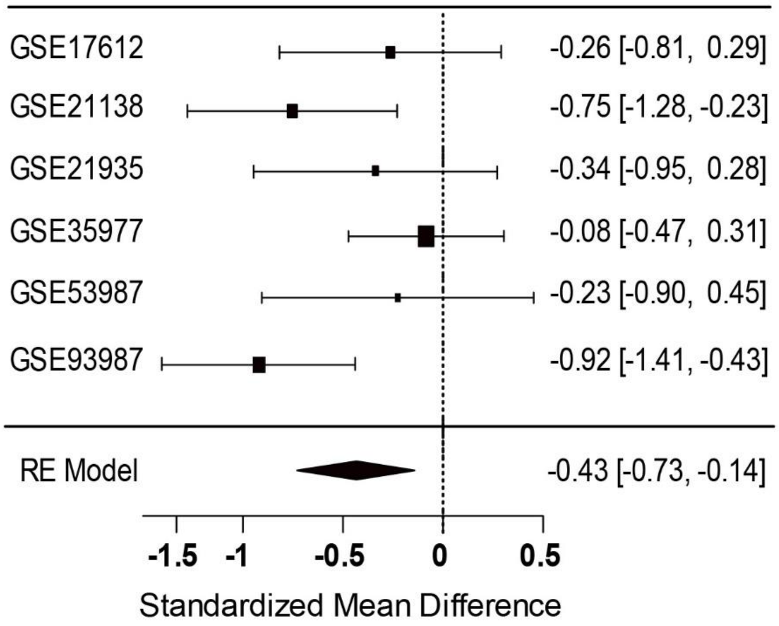

Supplementary Figure 4. A forest plot of differentially expressed genes in the postmortem PFC brain tissue from 286 schizophrenia patients and 343 nonpsychiatric controls from the *ATP5MD* meta-analysis, with the effect size and 95% confidence interval from each dataset and the pooled effect (random-effects (RE) model) from the meta-analysis.

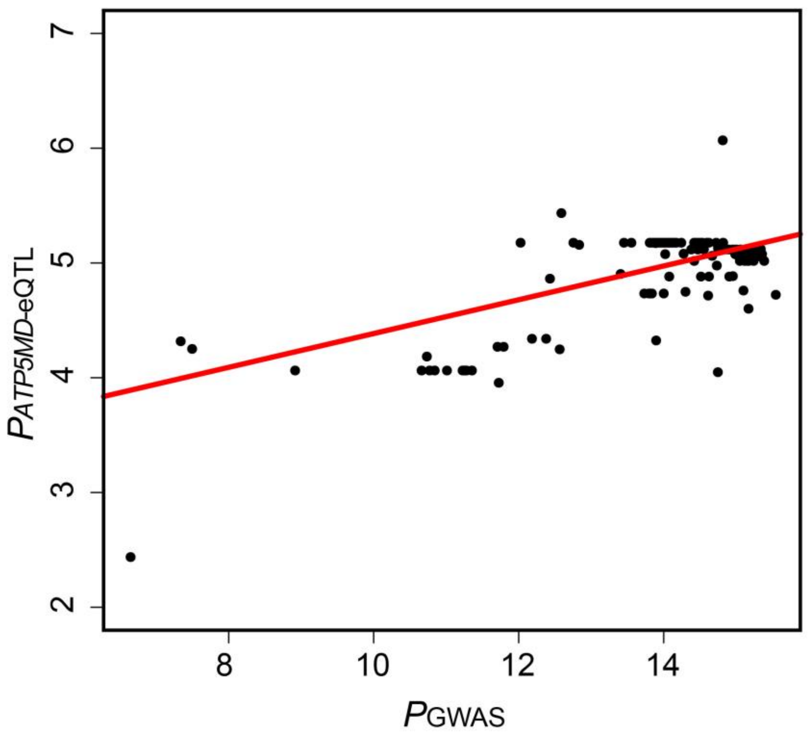

Supplementary Figure 5. Scatter plot for GWAS and *ATP5MD* eQTL *P* values of colocalized SNPs. SNPs with GWAS *P*<5e-8 and *ATP5MD* eQTL *P*<1e-3 were considered as colocalized SNPs. –Log10 transformed GWAS and eQTL *P* values were shown on x-axis and y-axis. Pearson correlation analysis were performed between –log10 transformed GWAS and eQTL *P* values. Significant positive correlation is identified (r=0.424, *P*=3.766e-07).

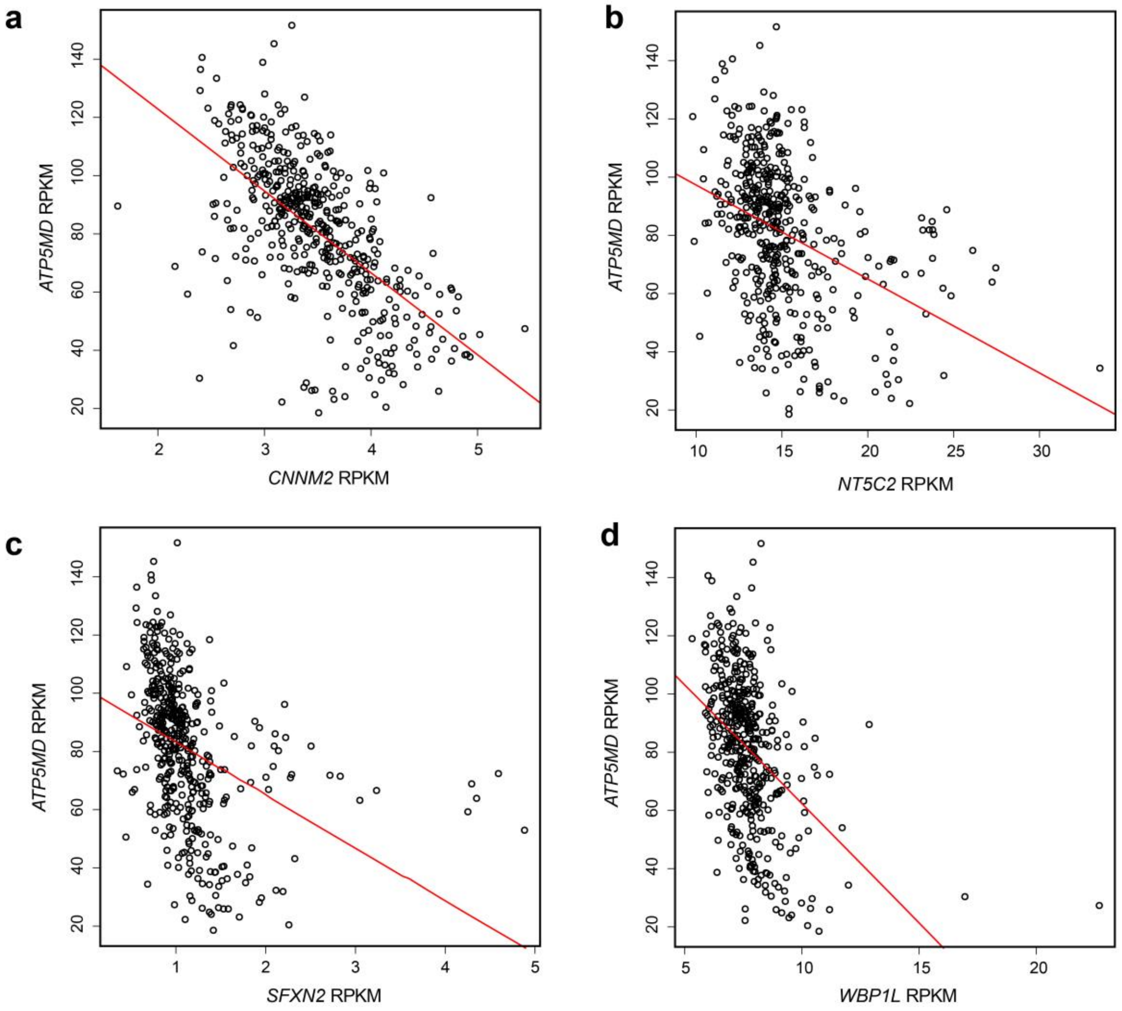

Supplementary Figure 6. Scatter plot for co-expression patterns of *ATP5MD* RPKM with *CNNM2* RPKM (**a**), *NT5C2* RPKM (**b**), *SFXN2* RPKM (**c**), *WBP1L* RPKM (**d**) examined with Pearson correlation analysis of RNA expression level from LIBD RNA-seq datasets.

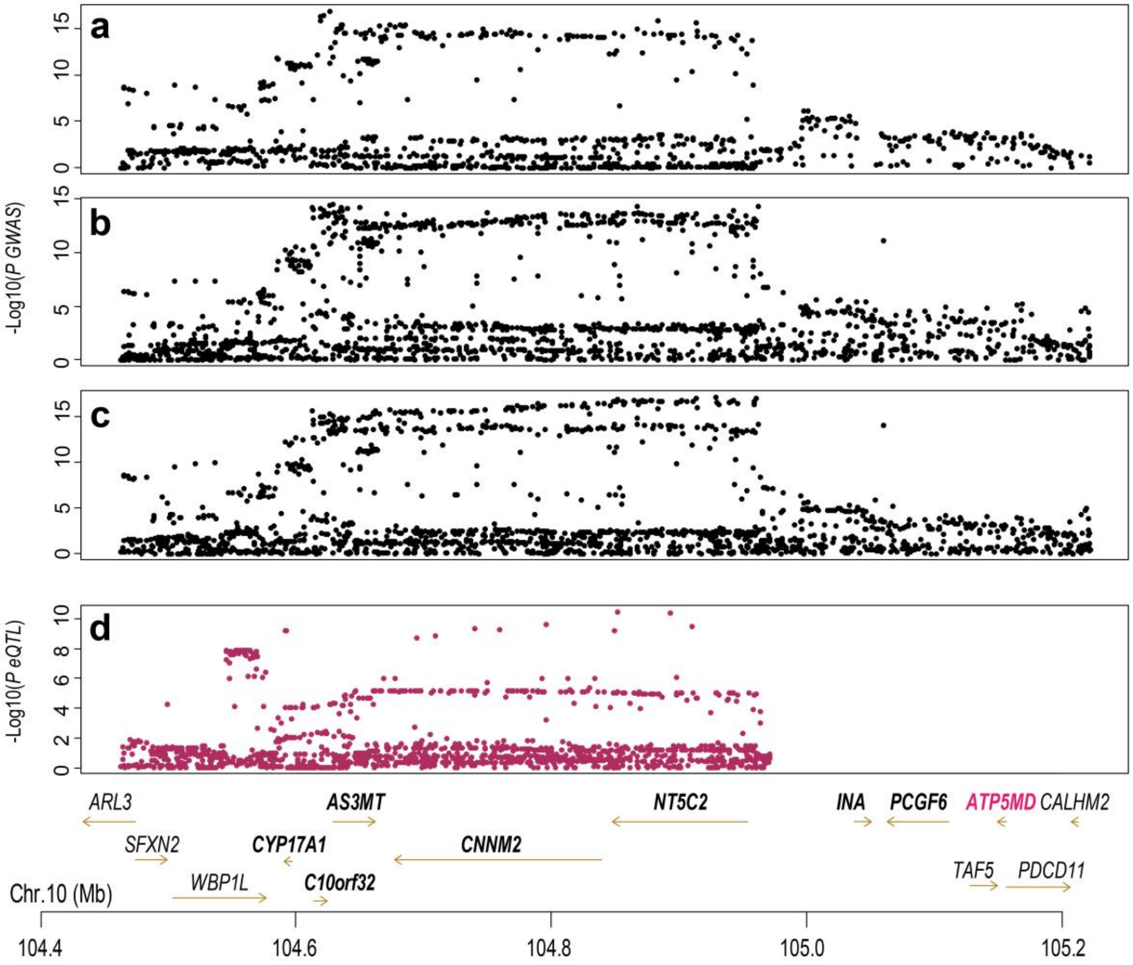

Supplementary Figure 7. Matched association patterns between schizophrenia GWAS and *ATP5MD* expression in chr10q24.32 were validated in three GWAS datasets including Chinese *Han* GWAS (**a**), PGC bipolar disorder and schizophrenia GWAS (**b**), and CLOZUK GWAS (**c**), as well as the LIBD PFC-eQTL dataset (**d**). Chromosome positions for each variant in the corresponding genes are shown on the bottom.

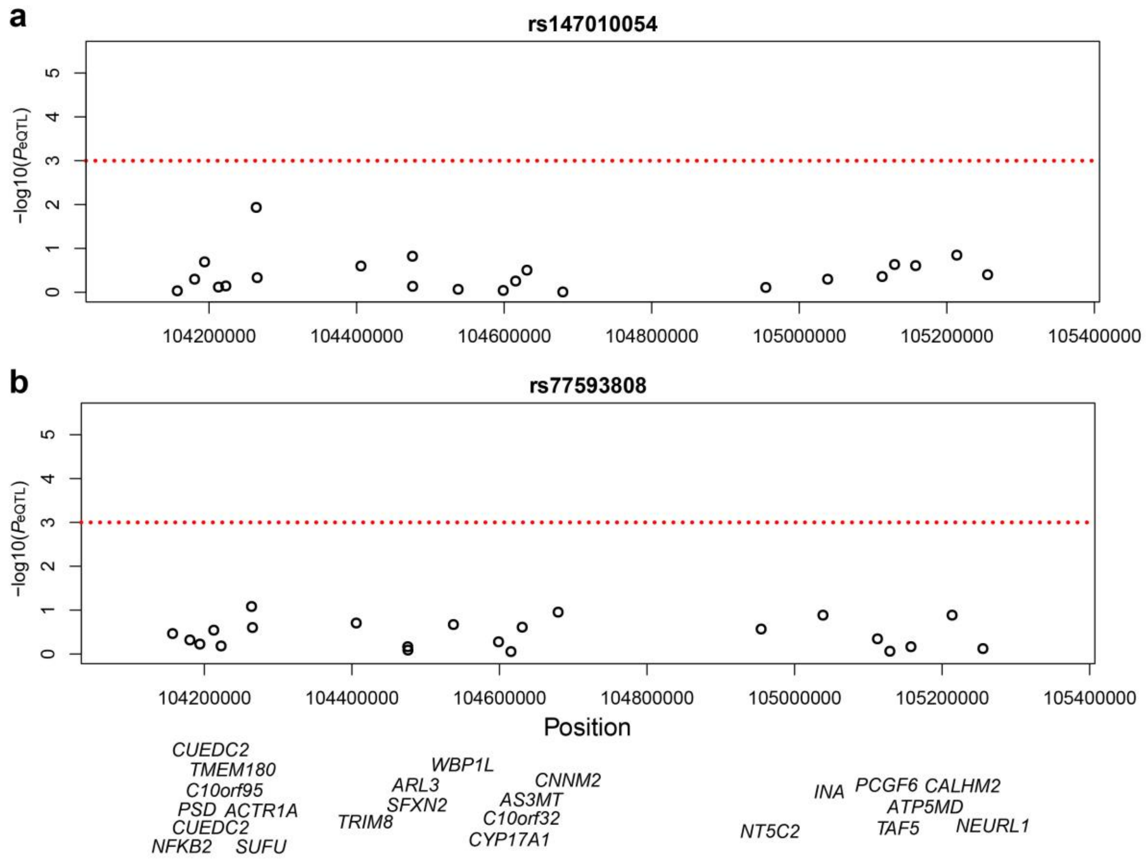

Supplementary Figure 8. Association of 10q24.32 index SNPs rs147010054 (**a**) and rs77593808 (**b**) genotype with gene expression in the 10q24.32 locus in the frontal cortex samples from the LFuN data set (n= 187). Locations of genes within the region are shown on the bottom. *P* values were calculated by MatrixEQTL. The red dash line represents as P_{eQTL}=1e-3.

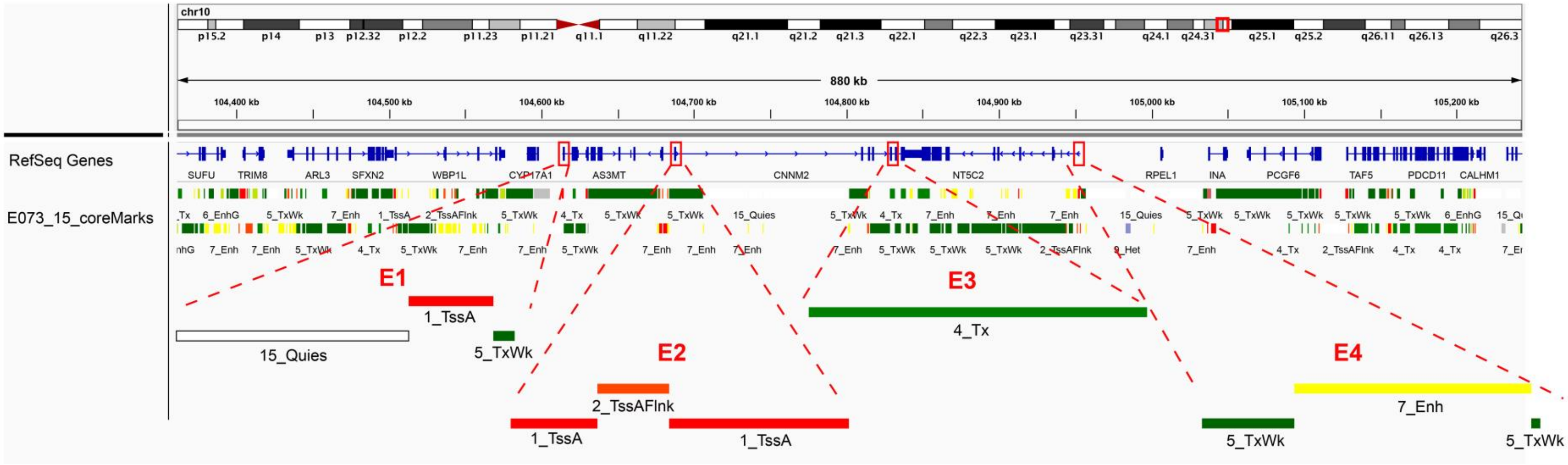

Supplementary Figure 9. Chromosome states of E1~E4. DLPFC derived chromosome state data were obtained from chromHMM. 1_TssA represents 'Active TSS'. 2_TSSAFlnk represents 'Flanking Active TSS'. 4_Tx represents 'Strong transcription'. 5_TxWk represents 'Weak transcription'. 7_Enh represents 'Enhancers'. 15_Quies represents 'Quiescent/Low'.

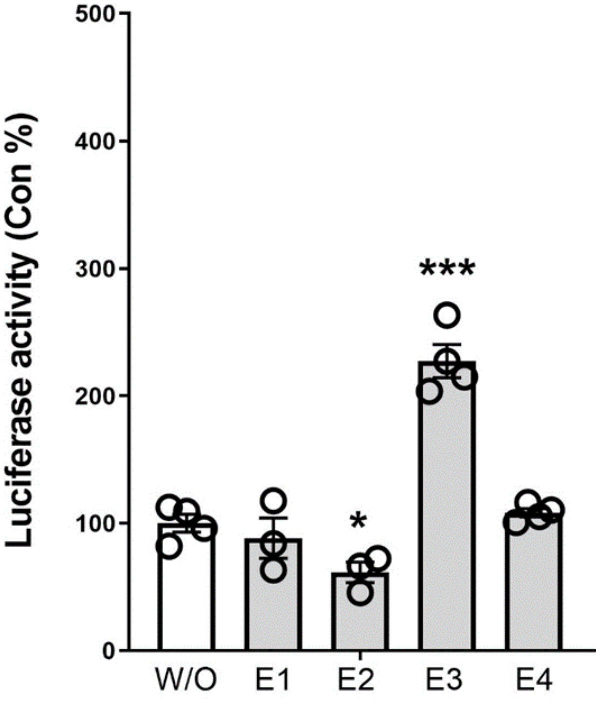

Supplementary Figure 10. Effects of the potential enhancer/silencer E1, E2, E3 and E4 regions on the *ATP5MD* promoter activities in SK-N-SH cells. For each enhancer/silencer region, an ~2 kb DNA fragments was cloned into the enhancer region in the pGL4.11 basic luciferase reporter driven by *ATP5MD* promoter. W/O, the reporter without an enhancer; E1~E4, the reporter with corresponding enhancer/silencer region. T-test was used to compare each group of E1~E4 with W/O (**P*<0.05, ***P*< 0.01 or ****P*<0.001). The data are shown as the mean ± SEM from at least three independent experiments with duplication.

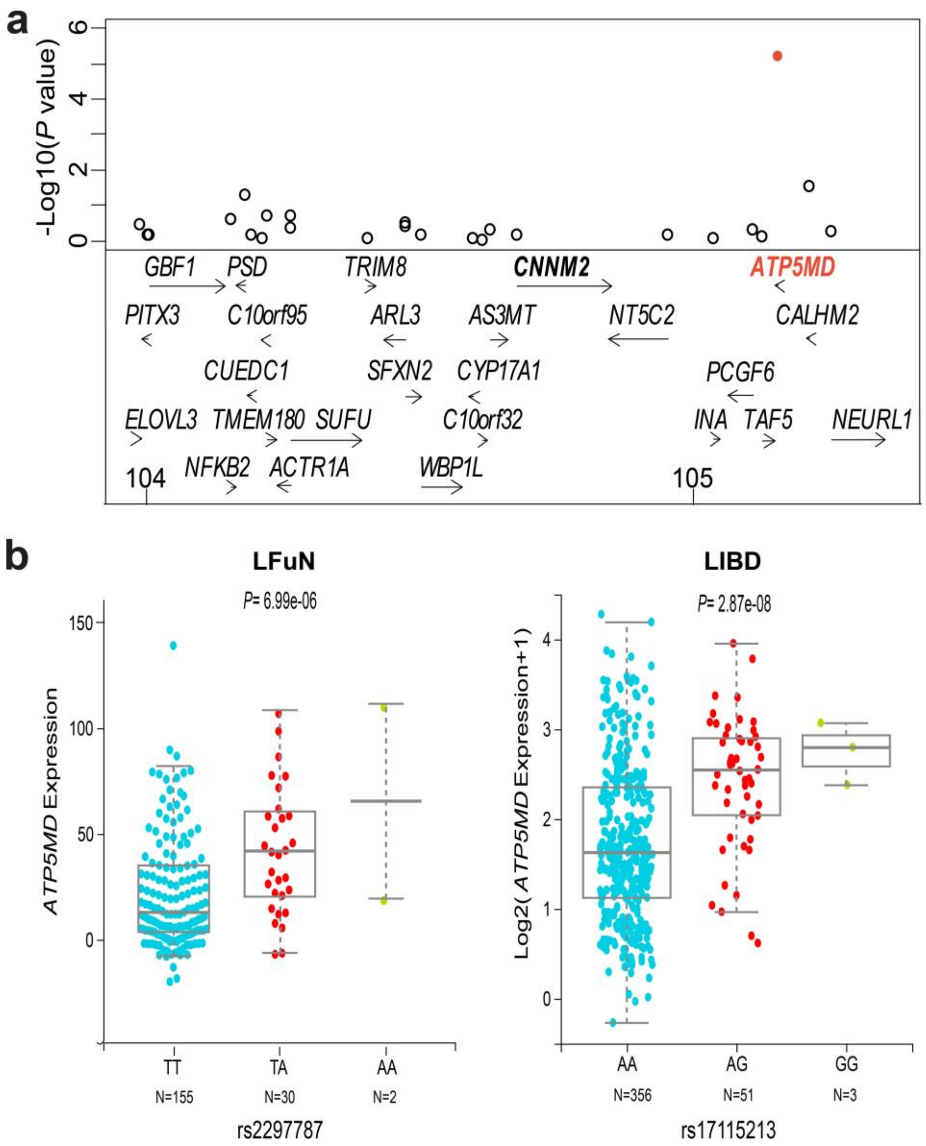

Supplementary Figure 11. (**a)** Association of the SNP rs2297787(T/A) genotype with gene expression in the 10q24.32 locus in the frontal cortex samples from the LFuN data set (n= 187). Locations of 25 genes within the region are shown on the bottom. (**b)** Association of the rs2297787 (**T/A**) genotypes with *ATP5MD* expression in the frontal cortex sample from the LFuN dataset (left, n= 187) and in the LIBD dataset (right, n=407). Since rs2297787 was not genotyped or imputed in the LIBD dataset, we therefore used its high LD SNP rs17115213 as a proxy readout (r^2=0.880 in Europeans). The middle line and the lower and upper edges of the box represent the median, first and third quartile of *ATP5MD* expression. *P* values were calculated by MatrixEQTL.

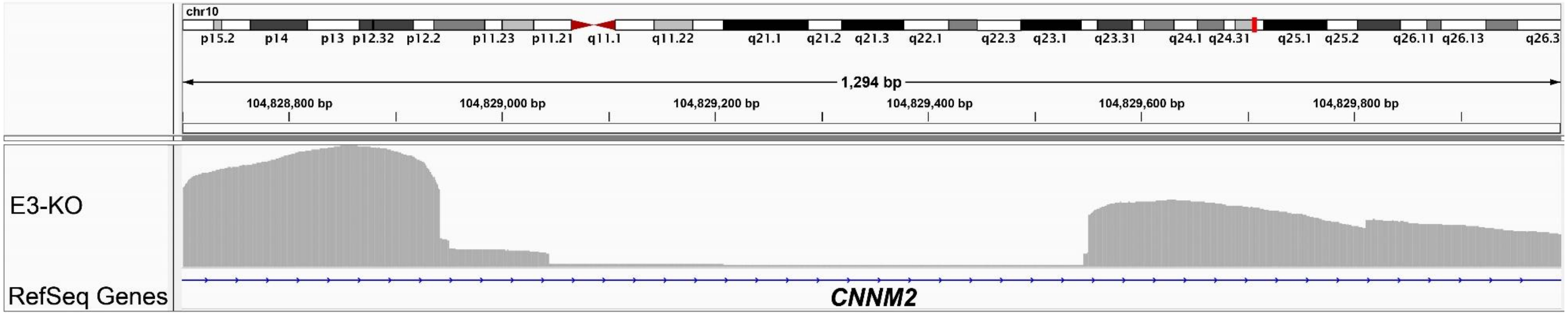

Supplementary Figure 12. Target sequencing results of DNA from E3-KO SK-N-SH cell lines. Bam file after mapping to reference genome was treated and visulaized with igv. For each position, the coverage was calculated and represented by peak height.

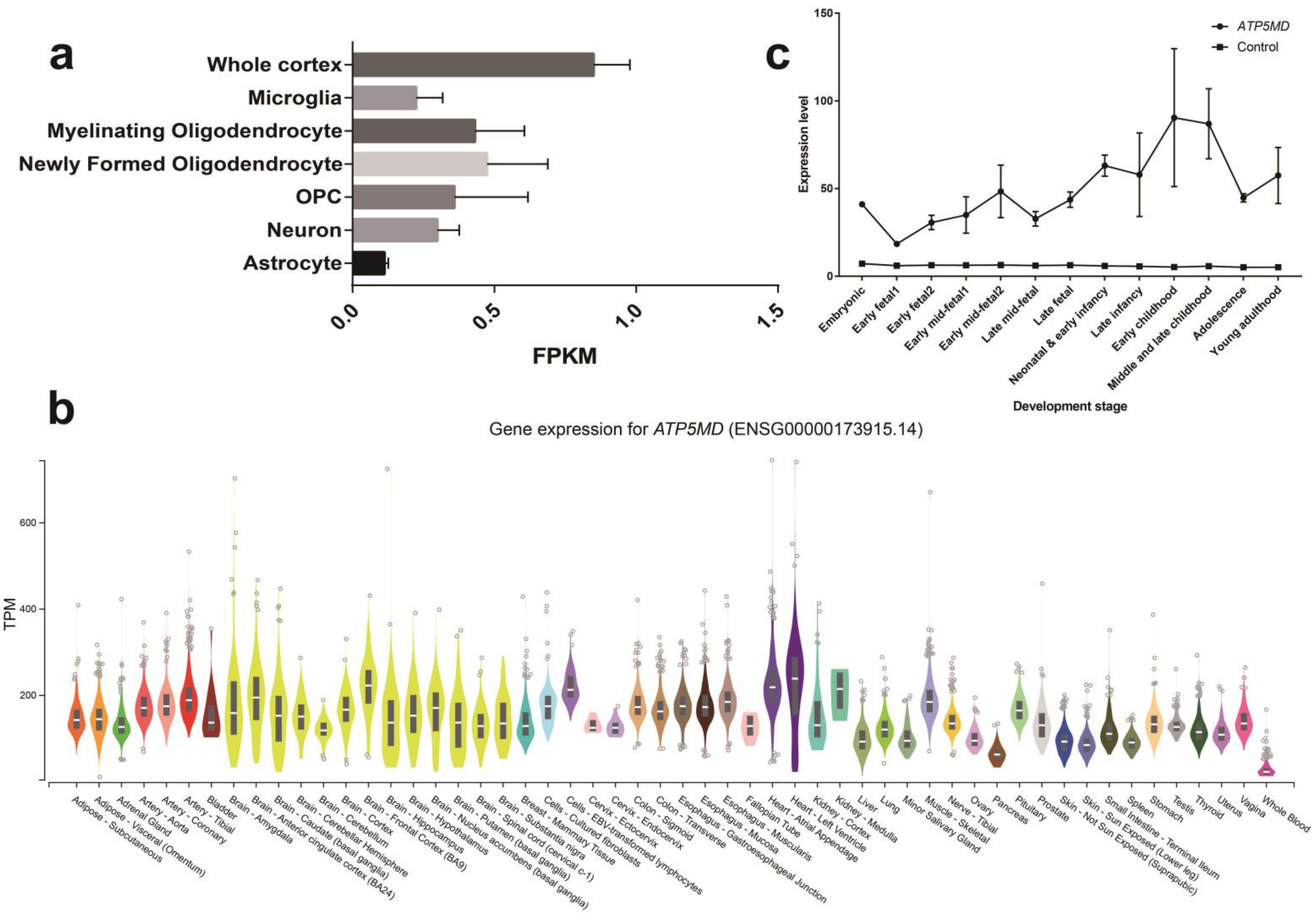

Supplementary Figure 13. Tissue or cell type expression pattern of *ATP5MD* from brain RNA-seq (**a**) and GTEx (**b**), and different developmental stages in the PFC region based on the BrainSpan data set (**c**).

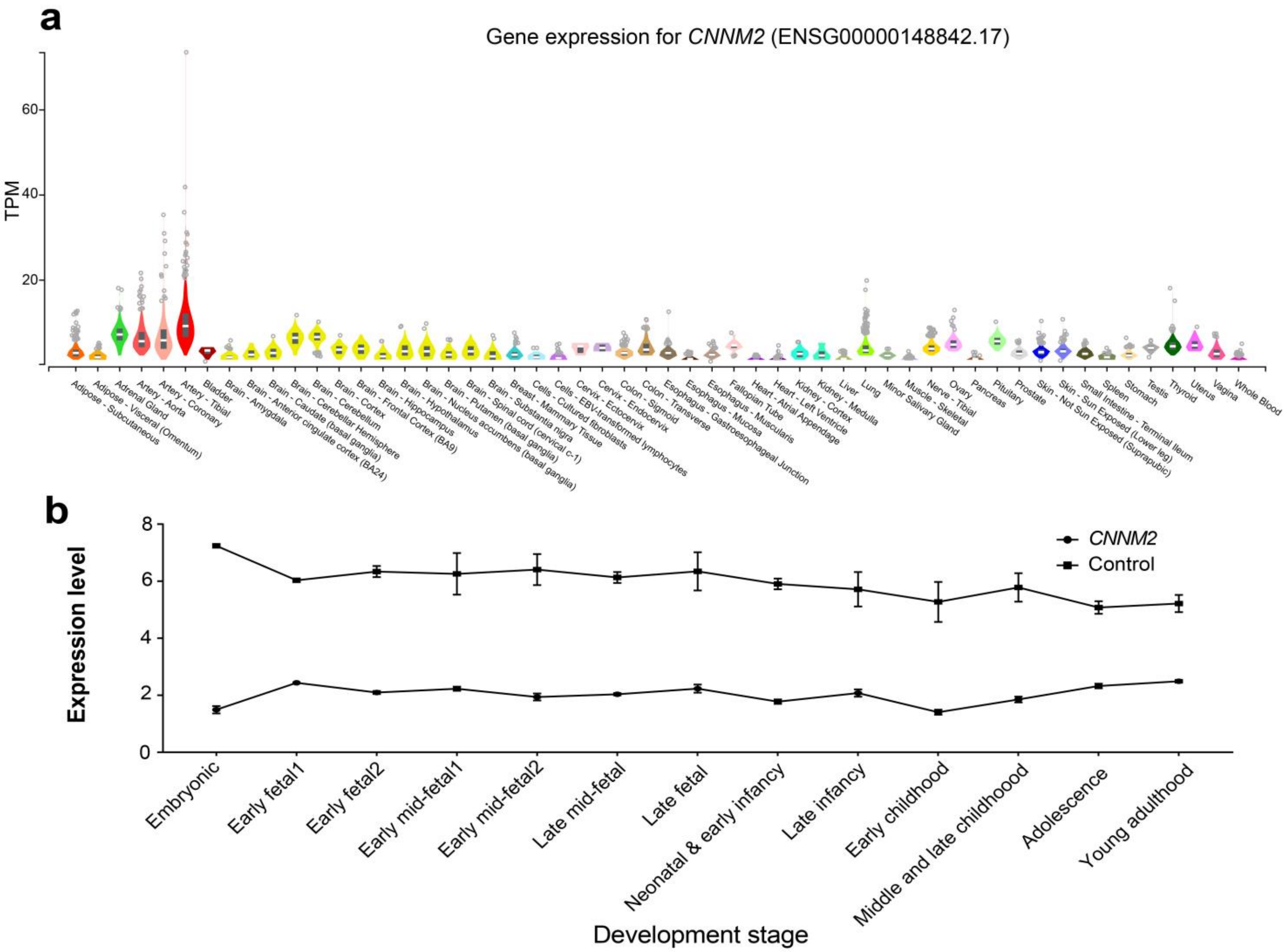

Supplementary Figure 14. Tissue expression pattern of *CNNM2* from and GTEx RNA-Seq (**a**), and different developmental stages in the PFC region based on the BrainSpan data set (**b**).

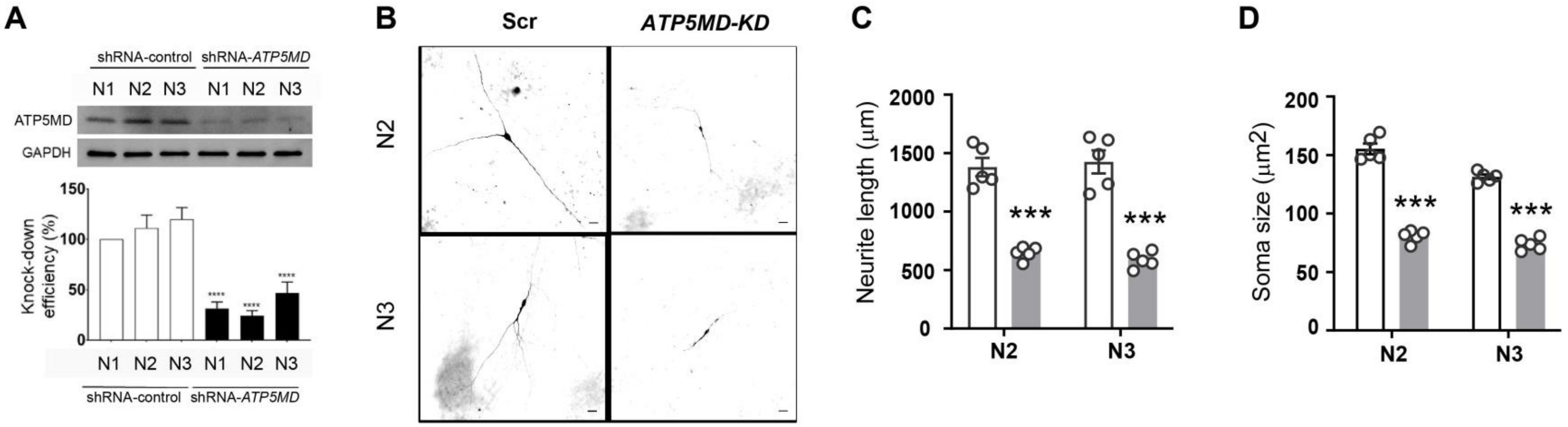

Supplementary Figure 15. (**a**) *In vitro* analysis of the efficacy of shRNAs against human *ATP5MD*. The expression construct for shRNA vector (shRNA-control) or shRNA-*ATP5MD* were infected into human iPSC-derived forebrain NPCs at day 0 of differentiation. After 72 hours, cell lysates were processed for Western blot analysis for ATP5MD and then replotted for GAPDH. Shown in (upper panel) is West Blot and in (bottom panel) is the quantification of the efficacy of three independent neurons, which were derived from the same iPSC (N1, N2 and N3). Values represent mean ± SEM (n = 3; ****p < 0.0001; ANOVA). (**b-d**) *ATP5MD*-KD attenuated neural development of iPSC-derived neurons. Sample projected confocal images (**b**) of 2-week-old human iPSC-derived cortical neurons with lentivirus-mediated coexpression of GFP and shRNA-control (Scr, white column) or shRNA-*ATP5MD* (grey column). Quantifications of soma size (**c**) and neurite length (**d**) were obtained from three independent iPSC-derived neuron samples (N2 and N3). The scale bar represents 20 μm.

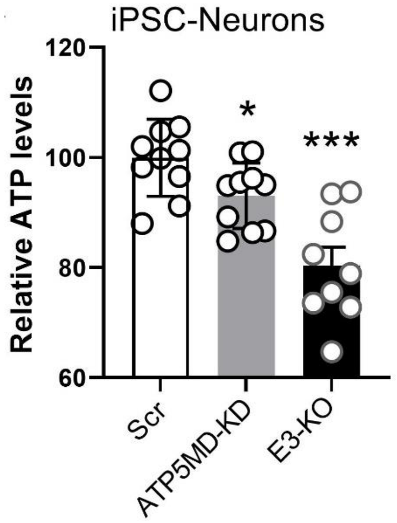

Supplementary Figure 16. Effects of *ATP5MD*-KD or *CNNM2*-E3 KO on the intracellular ATP levels in iPSC-derived neurons. Values represent mean ± SEM (*$p < 0.05$ and ***$p < 0.001$, two-tailed *t*-test.).

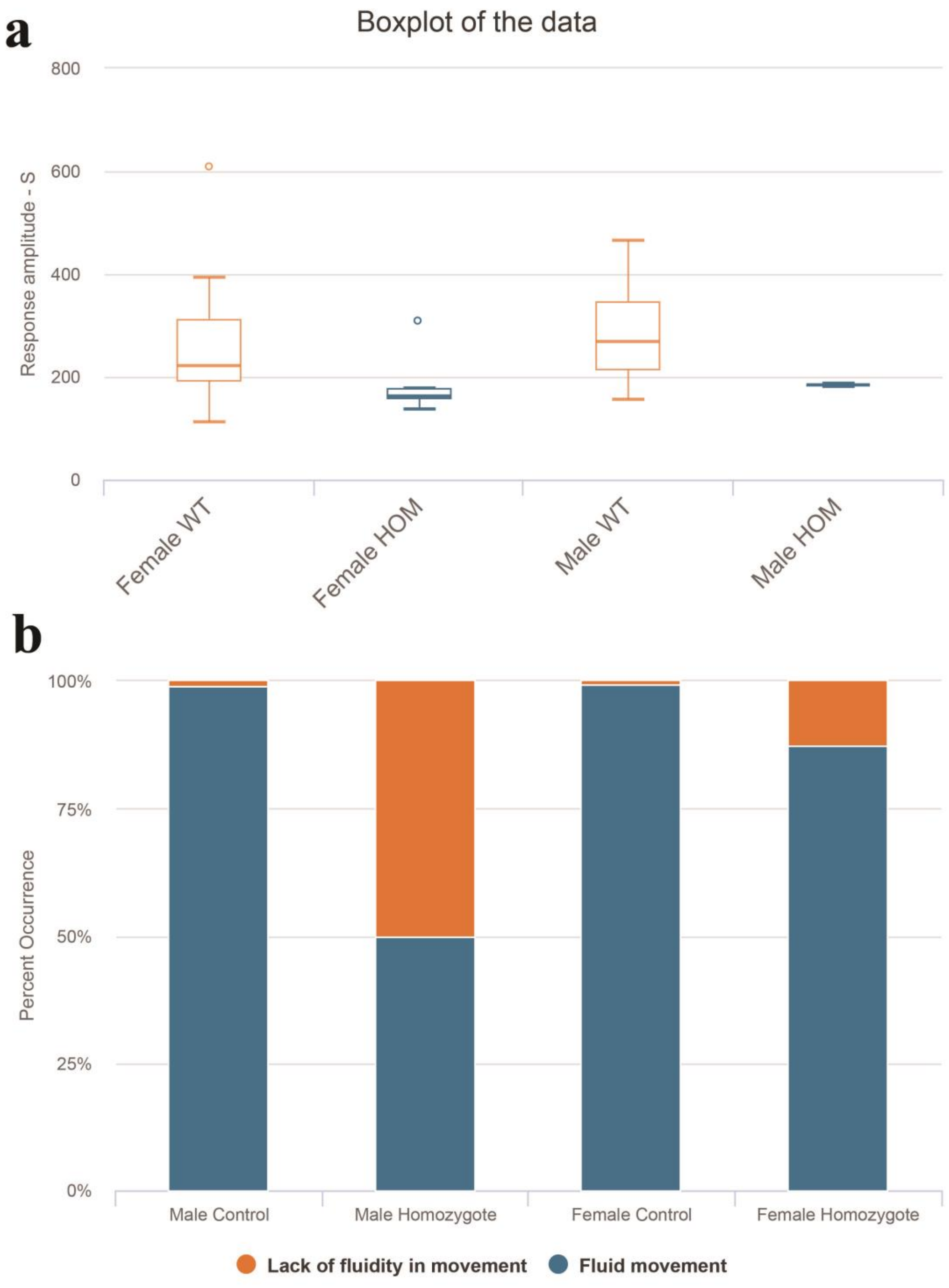

Supplementary Figure 17. Abnormal startle reflex (*P*=1.48e-34, **a**) and abnormal gait (*P*=1.32e-7, **b**) were observed for *atp5md* KO mouse from IMPC dataset.

Supplementary Tables

Supplementary Table 1. Enrichment results for genes located within 45 MAGMA identified novel loci with schizophrenia associated gene sets.

**Term**	**GeneRatio**	**BgRatio**	**Odds ratio**	***P***	***P*.adjust**	**count**
FMRP	10/90	794/20268	3.07	2.88E-03	1.44E-02	10
PSD	13/90	1395/20268	2.28	1.04E-02	2.59E-02	13
ARC_NMDAR	2/90	79/20268	5.81	5.00E-02	8.33E-02	2
de novo	1/90	51/20268	4.45	2.06E-01	2.58E-01	1
GABA	0/90	18/20268	0.00	1.00E+00	1.00E+00	0

Supplementary Table 2. Information of colocalized SNPs.

SNP	POS	A1	A2	P_{GWAS}	P_{eQTL}
rs17115100	104591393	T	G	1.5E-11	8.67E-05
rs1004467	104594507	G	A	2.28E-11	8.67E-05
rs3824755	104595849	C	G	1.76E-11	8.67E-05
rs138009835	104598995	A	G	5.81E-12	8.67E-05
rs7912206	104604563	T	C	1.26E-09	8.67E-05
rs11191416	104604916	G	T	1.02E-11	8.67E-05
rs150786824	104612941	A	G	5.5E-12	8.67E-05
rs112468006	104613020	A	G	4.62E-12	8.67E-05
rs3824754	104614350	T	C	6.21E-12	8.67E-05
rs4409766	104616663	C	T	1.92E-11	0.000066
rs11191425	104625970	T	C	2.04E-12	5.42E-05
rs11191426	104627230	T	G	1.68E-12	5.42E-05
rs7098825	104628234	C	T	6.82E-13	4.61E-05
rs17878846	104630412	T	A	4.37E-13	4.61E-05
rs77335224	104636276	T	C	2.84E-13	5.69E-05
rs3740393	104636655	C	G	3.32E-08	5.65E-05
rs11191434	104637508	C	T	3.87E-13	1.38E-05
rs3740390	104638480	T	C	4.1E-14	1.26E-05
rs72841270	104642237	G	T	4.79E-08	4.86E-05
rs144662567	104648482	C	CTT	1.94E-14	1.86E-05
rs11191447	104652323	T	C	1.6E-14	1.86E-05
rs74233296	104655350	C	T	1.66E-14	1.86E-05
rs35125602	104656039	A	G	1.66E-14	1.86E-05
rs113282265	104657248	G	A	1.04E-14	1.86E-05
rs12218148	104657469	A	C	1.52E-14	1.86E-05
rs11191453	104659852	C	T	1.37E-14	6.69E-06
rs11191454	104660004	G	A	1.33E-14	4.78E-05
rs10883798	104660688	G	A	1.37E-14	6.69E-06
rs17884001	104661245	T	C	1.35E-14	6.75E-06
rs4568943	104661881	A	C	1.36E-14	6.69E-06
rs17879819	104662215	T	C	7.03E-15	6.69E-06
rs12221193	104665267	C	A	1.5E-14	6.69E-06
rs185575325	104665997	G	C	1.84E-13	6.69E-06
rs77180047	104666757	A	G	9.85E-13	6.69E-06
rs7475853	104673097	A	G	9.14E-15	6.69E-06
rs12221064	104677126	T	C	1.52E-14	6.69E-06
rs2297787	104680137	A	T	1.53E-13	6.99E-06
rs17115213	104681143	G	A	1.19E-14	6.69E-06
rs77602510	104682602	A	G	1.31E-14	6.69E-06
rs78821730	104684544	A	G	1.19E-14	6.69E-06
rs12411886	104685299	A	C	1.03E-14	6.69E-06
rs10509759	104689665	T	A	1.12E-14	6.69E-06
rs112699822	104692633	A	C	1.17E-14	6.69E-06

rs112314091	104694654	A	G	8.38E-15	6.69E-06
rs145535890	104696990	A	ATGATAGCCC	3.68E-14	6.69E-06
rs5011520	104697516	A	G	2.92E-14	6.69E-06
rs11191472	104707016	T	A	8.63E-15	6.69E-06
rs10883806	104713076	T	C	1.47E-14	6.69E-06
rs11191474	104713113	C	A	1.62E-14	6.69E-06
rs11191475	104713162	T	C	1.5E-14	6.69E-06
rs12413409	104719096	A	G	9.96E-15	8.4E-06
rs10883808	104721126	T	A	1.02E-14	6.69E-06
rs78260931	104721962	C	G	9.16E-15	6.69E-06
rs11191479	104723620	C	T	1.01E-14	6.69E-06
rs11191484	104729249	A	G	9.43E-15	6.69E-06
rs145010450	104737697	TG	T	7.87E-15	6.69E-06
rs10883815	104739179	C	T	8.28E-15	6.69E-06
rs112390216	104741031	T	C	7.52E-15	6.69E-06
rs77860422	104748009	A	G	5.54E-15	8.38E-06
rs11191499	104764271	C	T	5.24E-15	0.000018
rs11191502	104765494	G	T	3.84E-15	6.69E-06
rs113554404	104766120	T	C	3.85E-15	6.69E-06
rs11191505	104766891	T	C	3.65E-15	6.69E-06
rs74444347	104769275	C	A	3.33E-15	6.69E-06
rs12221335	104769392	A	T	5.98E-15	6.69E-06
rs79059851	104769709	C	T	3.96E-15	6.69E-06
rs11191514	104773364	T	C	3.08E-15	6.69E-06
rs77787671	104776205	T	C	2.65E-15	6.69E-06
rs11191515	104776527	A	G	1.48E-14	6.69E-06
rs113970872	104778812	T	C	2.5E-15	6.69E-06
rs11191517	104780038	A	G	2.62E-15	6.69E-06
rs11191519	104784918	T	C	2.59E-15	6.69E-06
rs11191521	104789475	G	T	1.97E-15	6.69E-06
rs11191522	104789580	A	G	2.92E-15	7.65E-06
rs146748241	104792250	C	CAG	1.85E-15	7.65E-06
rs75970938	104793648	C	T	1.5E-15	7.65E-06
rs79668541	104793904	T	C	1.33E-15	7.65E-06
rs76752100	104794086	T	G	4.36E-15	7.65E-06
rs11191531	104806898	C	G	1.49E-15	7.65E-06
rs12219027	104811203	C	T	1.82E-15	7.65E-06
rs11191534	104815827	G	A	1.56E-15	7.65E-06
rs144441152	104815871	A	AAAAAG	1.86E-15	7.45E-06
rs11191543	104824387	A	G	3.55E-15	7.65E-06
rs3781285	104825665	G	C	1.17E-15	7.65E-06
rs147687891	104828026	G	GGAGGCCAAGACAGT	1.58E-15	6.69E-06
rs1926032	104829469	T	C	2.69E-13	3.71E-06
rs943037	104835919	T	C	9.11E-16	7.65E-06
rs12219901	104840967	G	A	1.05E-15	7.65E-06
rs10458729	104841479	T	C	1.04E-15	7.65E-06

rs11191548	104846178	C	T	1.87E-15	9.01E-05
rs10430665	104848430	T	C	9.38E-16	8.4E-06
rs10786736	104849116	C	G	4.56E-11	5.55E-10
rs11191551	104850835	G	A	8.72E-16	8.36E-06
rs17094683	104851301	T	G	7.68E-16	8.36E-06
rs12217501	104851889	C	T	4.79E-16	7.62E-06
rs12220743	104851912	T	C	5.26E-16	7.62E-06
rs12412038	104856162	A	G	5.02E-16	8.72E-06
rs140857253	104856764	C	CAA	2.24E-15	8.72E-06
rs11191555	104857523	C	A	5.38E-16	8.72E-06
rs11191557	104864614	G	C	5.48E-16	8.72E-06
rs11191558	104864678	A	G	5.49E-16	8.72E-06
rs11191559	104867686	T	C	9.27E-16	9.67E-06
rs11191560	104869038	C	T	6.26E-16	7.54E-06
rs12413046	104871204	G	A	6.03E-16	9.67E-06
rs10883832	104871279	G	T	4.3E-16	9.67E-06
rs9633712	104873761	C	G	7.09E-16	9.67E-06
rs79082900	104877302	C	T	7.06E-16	9.67E-06
rs11191564	104878543	T	C	7.42E-16	9.67E-06
rs1060240	104883337	G	A	3.97E-15	9.67E-06
rs10883835	104884208	C	T	8.05E-16	9.67E-06
rs11191568	104886374	A	G	8.29E-16	1.75E-05
rs732998	104897901	C	T	1.6E-15	8.59E-07
rs11191574	104898126	T	C	1.16E-15	1.31E-05
rs11191575	104898337	T	C	1.08E-15	8.46E-06
rs79993475	104901031	G	A	9.69E-16	8.81E-06
rs12220375	104901491	C	T	7.25E-16	8.81E-06
rs201779984	104902430	GA	G	5.03E-16	8.81E-06
rs11191580	104906211	C	T	7.07E-16	2.53E-05
rs79680647	104910959	A	G	6.19E-16	8.46E-06
rs11191582	104913653	A	G	2.97E-16	1.91E-05
rs74233809	104913940	C	T	2.54E-15	1.93E-05
rs12416331	104928914	A	T	4.59E-16	8.46E-06
rs11191587	104929716	C	T	1.05E-15	8.46E-06
rs12219304	104931584	C	G	8.76E-15	1.32E-05
rs11191593	104939215	C	T	1.07E-15	8.46E-06
rs79237883	104940946	C	T	9.41E-16	8.46E-06
rs34747231	104942244	G	T	5.27E-16	8.46E-06
rs77420391	104945823	A	G	3.19E-15	1.32E-05
rs12414028	104957629	A	T	1.96E-12	0.000111
rs112913898	104958900	A	G	1.3E-15	1.32E-05
rs11191607	104959188	T	G	2.48E-15	1.32E-05
rs113278154	104960464	T	C	1.93E-15	1.06E-05

Supplementary Table 3. Pearson correlation between *ATP5MD* and genes around.

**Gene.name**	**Gene.stable.ID**	**Chrom**	**Transcript Start Site**	**Strand**	**r**	***P***
SFXN2	ENSG00000156398	10	104503249	1	-0.38	5.98E-17
WBP1L	ENSG00000166272	10	104576021	1	-0.43	4.38E-22
CYP17A1	ENSG00000148795	10	104597290	-1	0.04	3.80E-01
AS3MT	ENSG00000214435	10	104661656	1	-0.03	5.62E-01
CNNM2	ENSG00000148842	10	104849978	1	-0.62	3.00E-49
NT5C2	ENSG00000076685	10	104953056	-1	-0.38	4.91E-17
RPEL1	ENSG00000235376	10	105007773	1	-0.03	4.82E-01
INA	ENSG00000148798	10	105050108	1	0.13	5.25E-03
PCGF6	ENSG00000156374	10	105110891	-1	0.12	1.37E-02

Supplementary Table 4. Oligonucleotide primers used in this study.

**A: Primers for generation of *ATP5MD* promoter constructs**	
*ATP5MD*_Pro_F	cggggtaccAAAGAAGTGATGACCGGGTG
*ATP5MD*_Pro_R	cccaagcttAATCCTTCCGTGCCTCTCTC

**B: Primers for generation of a series of Enhancer constructs**	
*ATP5MD*_V1_F	cgcggatccTGGGTTCTTGGTATCTTGCAAG
*ATP5MD*_V1_R	acgcgtcgacCTCTGGAAGCCTAAACTGCA
*ATP5MD*_E1_F	cgcggatccCATGGGAAAAGGAAGGGACG
*ATP5MD*_E1_R	acgcgtcgacCAGGCGTGATAGTGGGCA
*ATP5MD*_E2_F	ggaagatctAGTGAAGAAGGCTACGGGAC
*ATP5MD*_E2_R	acgcgtcgacGCCCCACATCATTCTCAACC
*ATP5MD*_E3_F	cgcggatccTCACCACTTACTCACTGCCC
*ATP5MD*_E3_R	ccgctcgagGTGCTGTGTGAATTCCGGTT
*ATP5MD*_E4_F	cgcggatccTGCCTGGCCGAGAATTCTAA
*ATP5MD*_E4_R	acgcgtcgacTGTGCCTCAAAAGCCATGAT

**C: Primers for point mutation of rs2297787 and rs1926032**	
rs201740017_MT_F	TGTTCTCATCACAAAAAAAAAGATAAATGTAAGAGG
rs201740017_MT_R	CCTCTTACATTTATCTTTTTTTTTGTGATGAGAACA
rs2297787_MT_F	GTAATTTCTGTGCATGACACTTATTTATTTaTTTTTTTTTGGTCCCGTGTGTGGGCCT
rs2297787_MT_R	AGGCCCACACACGGGACCAAAAAAAAAtAAATAAATAAGTGTCATGCACAGAAATTAC
rs1926032_MT_F	GTGTGCGCTCTGCCAAGCAGCaCCATCCCCAGAGGCCAGCAAG
rs1926032_MT_R	CTTGCTGGCCTCTGGGGATGGtGCTGCTTGGCAGAGCGCACAC

**D: Primers for generation of *ATP5MD* shRNA constructs**	
ATP5MD-shRNA_F	CCGGGAAGCATTGCATTGATTGTCTCTCGAGAGACAATCAATGCAATGCTTCTTTTTTG
ATP5MD-shRNA_R	AATTCAAAAAAGAAGCATTGCATTGATTGTCTCTCGAGAGACAATCAATGCAATGCTTC

E: Primers for generation of *ATP5MD* sgRNA constructs	
ATP5MD-sgRNA_F_F	CACCGGACTGGCGCTACGTGTCAGAGGG
ATP5MD-sgRNA_F_R	AAACCCCTCTGACACGTAGCGCCAGTCC
ATP5MD-sgRNA_R_F	CACCGTTCGAGCTATCAACCCCAATTGG
ATP5MD-sgRNA_R_R	AAACCCAATTGGGGTTGATAGCTCGAAC

F: Primers for generation of 4C libraries	
USMG5-4C_F	GTATCTTCTGTGCCCCTG
USMG5-4C_R	TTCGAAGCGGGGCTAAGGT
E3-4C_F	CGTGGGTAGGATTTCTCCT
E3-4C_R	CGCATACACAGATTTCCTGGGAAT

G: Primers for generation of *EMSA probes*	
rs1926032-C-F	GGCCTCTGGGGATGGCGCTGCTTGGCAGAGC
rs1926032-C-R	GCTCTGCCAAGCAGCGCCATCCCCAGAGGCC
rs1926032-T-F	GGCCTCTGGGGATGGTGCTGCTTGGCAGAGC
rs1926032-T-R	GCTCTGCCAAGCAGCACCATCCCCAGAGGCC

Restriction enzyme sites were shown in lower case in A and B.

Datasets

Dataset 1. Information of 5216 loci.

Dataset 2. Information of colocalized target genes of MAGMA identified loci by multi-SNP based SMR analysis.

Dataset 3. DEG analysis results of *ATP5MD* for CMC and LIBD cohort performed by Lieber Institute.
