# Supplementary material for: Downregulation by CNNM2 of ATP5MD expression in the 10q24.32 schizophrenia-associated locus involved in impaired ATP production and neurodevelopment
Source: NPJ Schizophr. 2021 May 21;7:27. doi: 10.1038/s41537-021-00159-y (PMC8139961; doi:10.1038/s41537-021-00159-y)
Supplement: Supplementary file 5 — Reporting Summary [file 41537_2021_159_MOESM5_ESM.pdf]

nature research

Corresponding author(s): Cunyou Zhao
Last updated by author(s): Mar 30, 2021

Reporting Summary

Nature Research wishes to improve the reproducibility of the work that we publish. This form provides structure for consistency and transparency in reporting. For further information on Nature Research policies, see our Editorial Policies and the Editorial Policy Checklist.

Statistics

For all statistical analyses, confirm that the following items are present in the figure legend, table legend, main text, or Methods section.

n/a	Confirmed	
☐	☒	The exact sample size (n) for each experimental group/condition, given as a discrete number and unit of measurement
☐	☒	A statement on whether measurements were taken from distinct samples or whether the same sample was measured repeatedly
☐	☒	The statistical test(s) used AND whether they are one- or two-sided<br>*Only common tests should be described solely by name; describe more complex techniques in the Methods section.*
☒	☐	A description of all covariates tested
☐	☒	A description of any assumptions or corrections, such as tests of normality and adjustment for multiple comparisons
☐	☒	A full description of the statistical parameters including central tendency (e.g. means) or other basic estimates (e.g. regression coefficient) AND variation (e.g. standard deviation) or associated estimates of uncertainty (e.g. confidence intervals)
☒	☐	For null hypothesis testing, the test statistic (e.g. F, t, r) with confidence intervals, effect sizes, degrees of freedom and P value noted<br>*Give P values as exact values whenever suitable.*
☒	☐	For Bayesian analysis, information on the choice of priors and Markov chain Monte Carlo settings
☒	☐	For hierarchical and complex designs, identification of the appropriate level for tests and full reporting of outcomes
☐	☒	Estimates of effect sizes (e.g. Cohen's d, Pearson's r), indicating how they were calculated

Our web collection on statistics for biologists contains articles on many of the points above.

Software and code

Policy information about availability of computer code

Data collection	All the data related to this manuscript are shown in main text and supplementary files.
Data analysis	There are no custom algorithms or softwares employed in our manuscript.

For manuscripts utilizing custom algorithms or software that are central to the research but not yet described in published literature, software must be made available to editors and reviewers. We strongly encourage code deposition in a community repository (e.g. GitHub). See the Nature Research guidelines for submitting code & software for further information.

Data

Policy information about availability of data

All manuscripts must include a data availability statement. This statement should provide the following information, where applicable:

- Accession codes, unique identifiers, or web links for publicly available datasets
- A list of figures that have associated raw data
- A description of any restrictions on data availability

All the data generated in this study were shown in the main text and supplementary files. Sequencing data for 4C assay have been uploaded to Gene Expression Omnibus with accession code GSE171077.

Field-specific reporting

Please select the one below that is the best fit for your research. If you are not sure, read the appropriate sections before making your selection.

☒ Life sciences ☐ Behavioural & social sciences ☐ Ecological, evolutionary & environmental sciences

For a reference copy of the document with all sections, see nature.com/documents/nr-reporting-summary-flat.pdf

Life sciences study design

All studies must disclose on these points even when the disclosure is negative.

Sample size	No clinical analysis was performed in this analyses. No statistical methods were employed predetermine sample size.
Data exclusions	No data were excluded from the analyses.
Replication	Yes
Randomization	No clinical analysis was performed in this analyses.
Blinding	DNA or RNA samples were randomly distributed in plates for RNA-sequencing, or qRT-PCR, and all assays were performed blind to diagnosis.

Reporting for specific materials, systems and methods

We require information from authors about some types of materials, experimental systems and methods used in many studies. Here, indicate whether each material, system or method listed is relevant to your study. If you are not sure if a list item applies to your research, read the appropriate section before selecting a response.

Materials & experimental systems

n/a	Involved in the study	
☐	☒	Antibodies
☐	☒	Eukaryotic cell lines
☒	☐	Palaeontology and archaeology
☒	☐	Animals and other organisms
☒	☐	Human research participants
☒	☐	Clinical data
☒	☐	Dual use research of concern

Methods

n/a	Involved in the study	
☒	☐	ChIP-seq
☒	☐	Flow cytometry
☒	☐	MRI-based neuroimaging

Antibodies

Antibodies used	This statement was described in the Methods section.
Validation	The source of antibodies employed in the study were reported in the Methods section.

Eukaryotic cell lines

Policy information about cell lines

Cell line source(s)	SK-N-SH, HEK 293-T
Authentication	There was no cell lines that were recently authenticated in this study.
Mycoplasma contamination	The cell lines have not been tested for mycoplasma contamination recently.
Commonly misidentified lines (See ICLAC register)	There was no commonly misidentified cell lines used in this study.
